# High-performance optical beam steering with nanophotonics

**DOI:** 10.1515/nanoph-2021-0805

**Published:** 2022-03-02

**Authors:** Sam Lin, Yixin Chen, Zi Jing Wong

**Affiliations:** Department of Materials Science and Engineering, Texas A&M University, College Station, TX 77843, USA; Department of Aerospace Engineering, Texas A&M University, College Station, TX 77843, USA

**Keywords:** beam steering, LiDAR, metasurfaces, nanophotonics, optical phased arrays

## Abstract

The ability to control and steer optical beams is critical for emerging technologies. Among these are light detection and ranging (LiDAR), laser display, free space communication, and single pixel imaging. Improvements in these areas promise enhanced 3D data collection capabilities, orders of magnitude increase in wireless data rate, less expensive cameras, and ever more immersive virtual/augmented reality (VR/AR) consumer electronics. Bulk mechanical or liquid crystal devices are conventionally utilized platforms that achieve optical beam steering, but they are bulky and limited in speed and reliability. Instead, chip-scale photonic platforms offer faster and more elegant mechanisms to manipulate light, capable of minimizing device size, weight, and power. Additionally, a critical device metric is its far field resolution, which influences fine feature detection in imaging applications, laser display quality, and signal power and fidelity of free space communication links. Strong light matter interaction achieved with nanophotonic approaches generally makes devices smaller and more efficient, yet ultimately these effects must be scaled to suitable aperture sizes to maintain good resolution. Recent years have seen rapid development in these performance characteristics, spurred by research on active metasurfaces, slow light waveguides, and waveguide phased arrays, with different architectures encountering unique tradeoffs between device complexity, resolution, and speed, in attempting to achieve groundbreaking values for all three. We review these diverse emerging nanophotonic approaches that aspire to achieve high-performance optical beam steering.

## 1 Introduction

Changing the direction of light at high speed is an objective important for a myriad of applications [1, 2]. Among these, LiDAR is a necessary tool for future autonomous navigation, as depth information gives artificial intelligent systems a leg up in object detection tasks [3]. It is also an excellent method of acquiring bathymetric data for scientific research [4] or biometric authentication [5]. Free space telecommunication would be boosted by the high carrier frequency of near infrared (NIR) light, enabling data rates as high as those supported by optical fibers, and benefit from well directed signal intensity [6]. Additionally, display technologies that currently rely on mirrors that flip back and forth could become even more portable, robust, and bright, and operate at a higher frame rate. Common to all these technologies are the requirement for high speed, high resolution, and minimized device size, weight, and power.

The task of redirecting a laser beam becomes more challenging as commercial applications demand faster, smaller, and more efficient devices. Conventional methods are typically mechanical or liquid crystal based [7]. For example, rotating polygonal mirrors are commonly found in barcode readers, and digital micromirror devices are the basis of laser projectors [8]. Higher power lasers may use gimbals, lenslet arrays, or Risley prisms to reorient [9]. The large refractive index response in liquid crystals has been thoroughly exploited [10], [11], [12]. However, bulk mechanical systems are constrained by their size, and as a result speed, and typical response times for liquid crystals are only on the order of milliseconds [10, 13]. Alongside these have developed other more exotic methods for beam steering. Electrowetting prisms which bend light by reorienting a liquid surface were explored [14, 15], and electro-optic and acoustically modulated bulk materials provided a simple way to deflect light at high speed [16, 17], albeit with low modulation efficiency. Instead of relying on larger devices or contending with low speed, a better solution is to work towards electrically tuned solid-state phased array beam steering without moving parts, which circumvents all previous issues described. Nanophotonics, the science that explores interactions between light and nanoscale matter, has produced breakthroughs in imaging, sensing, and communication [18]. Devices based on nanophotonics are becoming the natural successors to the previous generation of beam steering devices, with small device volumes achieving ultrahigh-speed operation.

Nevertheless, antennas fashioned from nanophotonic approaches must be arranged into some array configuration. The phase of each antenna must be individually or collectively controlled to form a phased array. Thus, nanophotonics must also contend with tight integration and operational complexity to achieve high resolution. In recent years significant progress has been made to achieve beam steering of ever improving performance parameters, and novel phased array architectures with different physics and tradeoffs have been explored in conjunction with the continued development of integrated optical phased arrays [19]. To gain a broader understanding of the progress in achieving these objectives, an overview of the state of the art in a diverse set of beam steering technologies is in order.

In this review, we address broad classes of nanophotonics based device architectures utilized for active beam steering and evaluate their respective performance parameters. In the first section, we introduce the design requirements of beam steering devices and overview the physical constraints that limit them. The second section reviews the progress in designing active phase gradient metasurfaces constructed from subwavelength optical antennas. The third section inspects the work done on slow light beam scanning devices, a class of devices that leverage the low energy velocity of light to produce large photon momentum shifts. The fourth section explores the class of directional light couplers known as optical phased arrays (OPAs), integrated photonic structures that are wavelength- and/or phase-tunable. We summarize with an outlook for active beam steering in general, evaluate the prospects of each device class, and overview the necessary breakthroughs required for their commercialization.

## 2 Performance parameters for active beam steering

Despite the wide variety of beam steering platforms being researched, any platform must obey several physical principles to meet the performance metrics necessary for real world applications. Specifically, a steered beam should have a narrow beam width and be steerable across a large majority of a semicircle (1D scanning) or a hemisphere (2D scanning). The range over which the beam can be directed is referred to as its field of view (FOV). Further, the emission angle should be reconfigurable in real time at high speed with minimal radiation loss into other directions, which commonly manifest as undesirable side lobes.

The far field pattern (FFP), $F\left(\vec{\xi }\right)$ , of a beam steering device can be determined from its electromagnetic near field $E\left(\vec{r}\right)$ via the near field’s Fourier transform:

(1) $$F\left(\vec{\xi }\right)=\iint E\left(\vec{r}\right)e^{ik_{0}\left(\vec{r}\cdot \vec{\xi }\right)}d^{2}\vec{r}$$

Here $\vec{\xi }=\left(\phi ,\theta \right)$ are the latitude and longitude directions, and $\vec{r}=\left(x,y\right)$ is the position on the phased array plane. $k_{0}$ is the free space wavenumber. Assuming the near field of each antenna, $u\left(\vec{r}\right)$ , is identical, and we can write the near field as

(2) $$E\left(\vec{r}\right)=\sum_{n}C_{n}u\left(\vec{r}-{\vec{r}}_{n}\right)=u\left(\vec{r}\right)*\sum_{n}C_{n}\delta \left(\vec{r}-{\vec{r}}_{n}\right)$$

The coefficients $C_{n}$ encode the emitters’ phases and amplitudes and are therefore complex valued, and ∗ denotes convolution. $\delta \left(\vec{r}\right)$ is the 2D delta function. In accordance with standard antenna theory, the far field profile of optical phased arrays is the product of the FFP of individual elements and the FFP of an identical array with isotropic antennas, the array factor [20], [21], [22]:

(3) $$F\left(\vec{\xi }\right)=U\left(\vec{\xi }\right)\sum_{n}C_{n}e^{ik_{0}{\vec{r}}_{n}\cdot \vec{\xi }}$$

By nature of the Fourier transform (Eq. (1)), one can see that modulating the near field $E\left(\vec{r}\right)$ with a plane wave $e^{ik_{0}\vec{r}\cdot \vec{k}}$ corresponds to a shift of the far field pattern by $\vec{k}$ . Thus, a peak previously present at the origin can be moved to an arbitrary angle. This is the basis of phased array beam steering: the plane wave modulation corresponds to phasing individual emitters, i.e. generating a phase gradient.

**Figure 1:**
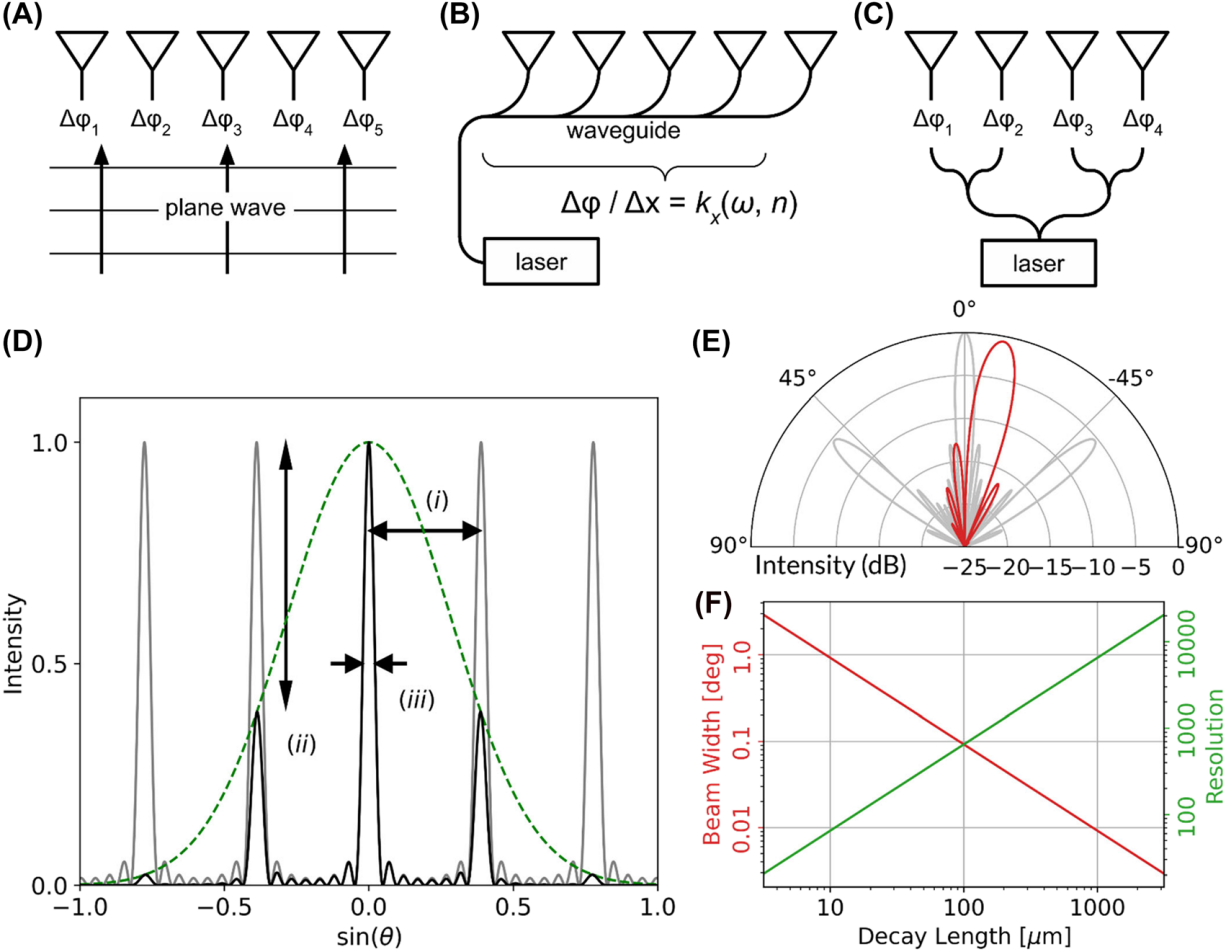
Principles of beam steering with nanophotonics. (A)–(C) Schematics of optical power routing in (A) active metasurfaces, (B) slow-light waveguides and frequency tuned gratings, and (C) integrated optical phased arrays. In (A)–(C), Δφ , dφ/dx , and $k_{x}\left(\omega ,n\right)$ denotes the phase change of each antenna, the spatial phase gradient, and wavenumber, respectively. The wavenumber depends on frequency and material permittivity. (D) shows an example of a typical 1D optical phased array far field pattern (FFP) (solid black line), where (i) indicates the usable field of view, constrained here by diffraction lobes on either side of the main lobe, (ii) shows the side mode suppression ratio (SMSR) quantified here by the ratio of main lobe power to side lobe power, and (iii) shows the beam width. The dashed green line shows the emission pattern of a single antenna, enveloping the array’s far field diffraction pattern (solid grey line). In (E), the red (grey) plot illustrates the FFP of an 8-antenna array with antenna spacing smaller (larger) than half wavelength, showing undesirable grating lobes produced by an insufficiently compact array. (F) For grating-like beam steering (B), the lower right plot shows at example of the longitudinal divergence angle and resolution versus decay length at 1550 nm with 60° FOV.

For antennas with uniform amplitude in a 1D uniformly spaced array, the array factor can be written as $AF∼\sin\;c\left[\frac{1}{2}N\left(ka\;\sin\left(\phi \right)\right)\right]$ for small ϕ , where *a* is the inter-element spacing [20]. This illustrates the important consequence that the beam width, typically defined as the full width half maximum (FWHM) of the dominant lobe in the far field, scales inversely with the physical length of the array for a given wavelength. The array size is interchangeably called the aperture size. This fundamental limit arises from the wave nature of light and cannot be circumvented by choice of materials. The beam width is a critical parameter for imaging techniques such as time of flight (TOF) since a large beam width decreases the imaging resolution of the 3D scene. For free-space telecommunication, a wide beam width increases the chance of third-party eavesdropping. The array size is fixed for architectures like active metasurfaces (Figure 1A) and integrated optical phased arrays along the array dimension (Figure 1C), while grating-like devices (Figure 1B), with optical power coupled from an in-plane source, have a near field profile characterized by exponential decay in $C_{n}$ along one spatial direction. In these cases, Eq. (1) dictates an inverse relation between the characteristic propagation length and the far field beam width [23], as plotted in Figure 1F. It is noted that while the mechanism illustrated in Figure 1A (Figure 1C) is exclusive to the devices in Section 3 (5), devices with a grating-like mechanism are present in both Sections 4 and 5.

The combined requirements of narrow beam width and large FOV provide a powerful figure of merit to evaluate the performance of beam steering devices. The resolution *N*, given by the ratio between the FOV and the beam width, counts the total number of distinguishable points that the beam steering device can project into the far field. This figure of merit is particularly important for imaging and display applications, where devices are benchmarked directly on the number of displayable pixels in the field of view.

For periodic arrays of antenna elements, the array period is an important design parameter. According to Fraunhofer diffraction theory, a half wavelength period guarantees radiation into a single lobe in the far field, whereas periodic antenna arrays with longer periods suffer energy loss from diffraction into side lobes, illustrated in Figure 1E. These unwanted lobes introduce noise to imaging and TOF measurements and again allow eavesdropping on telecom signals. Furthermore, the diffraction orders compete for space in the far field, reducing the effective FOV (Figure 1D), and decreasing resolution.

The side mode suppression ratio (SMSR) quantifies the emitted far field pattern quality and can be interpreted in several ways. For a theoretical sub-half-wavelength uniform array of point emitters, the SMSR quantifies the suppression of intensity maxima very close to the main beam, which arises from the spatial frequency introduced by the large-scale array profile. Likewise, for few-wavelength-spaced uniform arrays SMSR may also refer to the suppression of diffraction orders. In the case of nonuniform arrays, which mitigate side lobe formation, SMSR quantifies the impact of spurious lobes that arise from the side lobes’ redistributed intensity. Inadequate SMSR reduces the effective FOV and therefore device resolution.

Additionally, beam steering designs should also consider a device’s operating energy, dictated by the material platform. Typical thermo-optic phase modulators consume on the order of 10 mW per waveguide [24, 25], a consequence of resistive heating-based operation. However, other phase modulation strategies such as carrier injection modulation and electro-optic modulation are much more efficient, having switching energies on the order of picojoules [26]. These switching energies are derived from the energy required to charge an equivalent capacitor and thus scales with capacitance and device size. Operating energy also depends on the energy velocity of light: lower group velocities facilitate a large phase response with lower modulation depth.

Optical beam steering devices have several physical speed constraints. High speed integrated devices that operate with in-plane energy injection are ultimately limited by the speed of light, which limits the transfer of information over 1 mm to time delay of (300 GHz)^−1^. The available bandwidth is further reduced by the group index, which is typically on the order of 3, but may be higher than 20 for slow light devices. In practice, most devices operate far below this theoretical limit. A large majority of OPAs benchmark from 1–100 kHz, since they employ thermo-optic phase modulation, with response times limited by the thermal diffusivity of the device. On the other hand, active metasurfaces tuned via carrier injection may be much faster depending on the electrode conductivity and device capacitance. Faster still is electro-optic modulation, capable of achieving >100 GHz switching speeds.

The above considerations are reflected in the technical requirements of practical devices. An often-cited application of optical beam steering is LiDAR due to the growing demand for autonomous vehicle technology. Current and typical objectives of this type of sensor includes maintaining operation at over 30 frames per second, with a wide-angle FOV over 120° × 90°, and with beam width narrow enough to enable resolution along a single direction higher than 10^3^ [9, 27, 28]. As another example, a display beam steering devices must achieve >25 dB SMSR to attain sufficient black levels. Further, devices must conform to allotted power budgets, with handheld devices imposing the most stringent limits.

Nanophotonics provides a suitable platform to achieve these performance metrics, owing in part to its intrinsic length scale. Facilitated by advances in nanofabrication techniques and the discovery of novel materials, large optical effects can be achieved in very small active volumes by confining optical energy into the modulated materials. Consequently, modulator-like devices can attain drastically lower switching times through decreased bulk heat capacity and electrostatic capacitance. Similarly, in smaller devices the required operation energy is reduced. Leveraging nanophotonics has also provided for a path to attain sub-micron antenna spacing via tight chip-scale integration, which enhances phased array operation by preventing side lobes and increasing SMSR. The remainder of this paper discusses techniques used to create nanophotonic devices that push closer toward the fundamental limits of optical beam steering technology.

## 3 Active phase gradient metasurfaces

First, we explore recent progress in the field of active phase gradient metasurfaces. Phase gradient metasurfaces are arrays of subwavelength optical elements acting as antennas that apply a spatially varying phase shift to an incident plane wave. The geometry and material characteristics of each element determines the output phase at each point on the metasurface, allowing for control of the fundamental properties of light, including polarization, phase, amplitude, and chirality. The versatility of these devices has enabled researchers to construct micron thick lenses and beam deflectors [29], [30], [31]. However, the transmission and reflection characteristics of traditional metasurfaces are necessarily fixed at fabrication, limiting their applicability to dynamic beam steering and shaping [32, 33]. Considering this limitation, significant efforts have been expended to realize dynamically modulated phase gradients.

In recent years, transparent conducting oxides (TCOs) have attracted considerable attention due to the large electrical tunability of their refractive index. By applying an electrical bias across thin films of TCOs such as indium tin oxide (ITO), charge carrier accumulation can be induced near its interface with an insulator, locally changing the plasma frequency and therefore optical permittivity [34]. Additionally, these materials are attractive due to both their compatibility with the silicon photonics material platform and their stable operation across a large range of temperatures.

**Figure 2:**
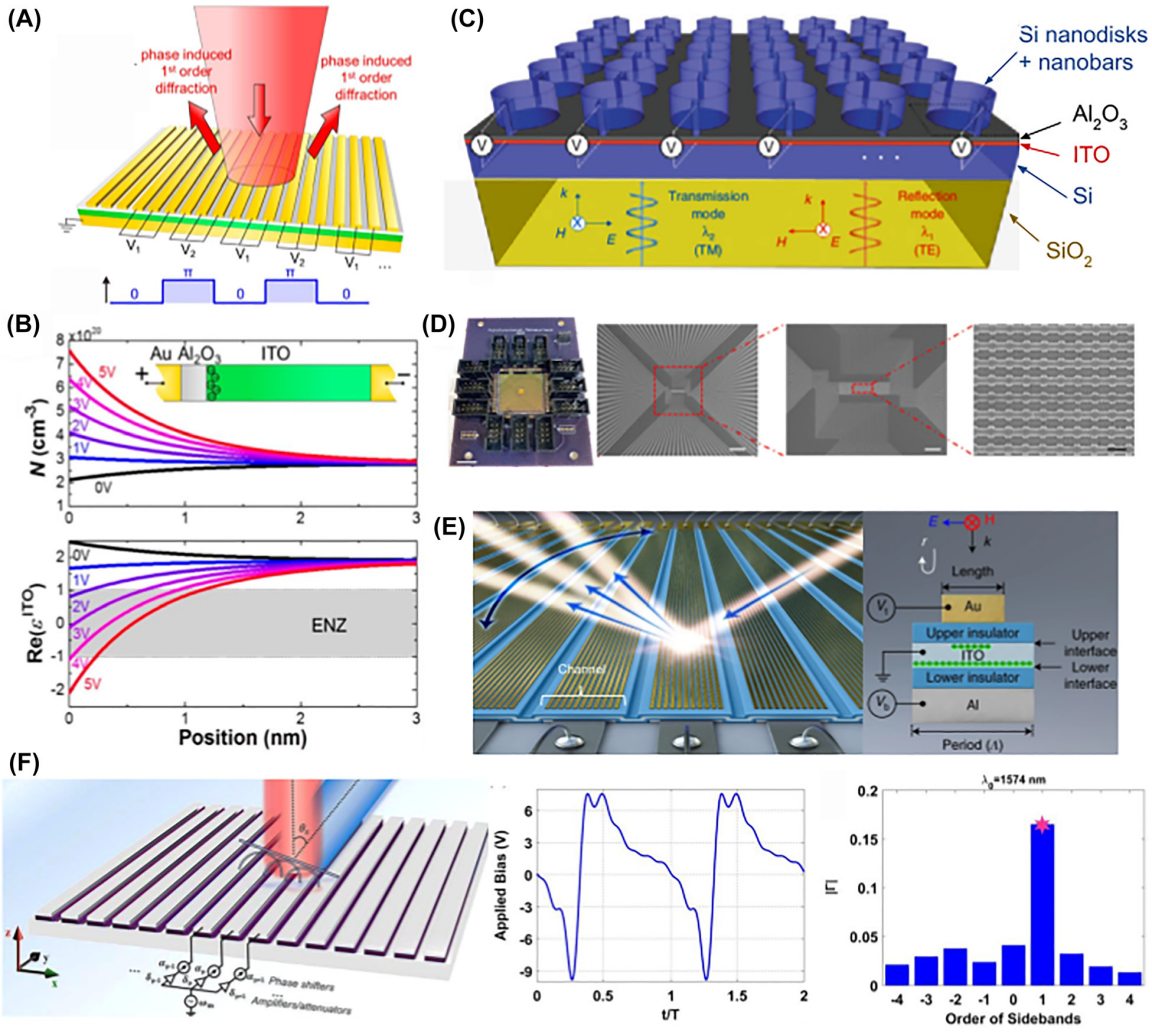
Active metasurfaces for beam deflection. (A) Diffraction switching through periodic step-biasing [35]. (B) The field-induced carrier concentration and permittivity change as a function of distance from the Al_2_O_3_-ITO interface. The inset illustrates the metal–insulator–metal structure for transparent conducting oxide antennas [35]. Adapted with permission from [35]. Copyright 2016 American Chemical Society. (C) An all-dielectric tunable metasurface with doped Si antennas and back plate [39]. Continuity is provided by the nanobars running along nanodisk rows. Adapted from [39] / CC BY 4.0. (D) A fishbone-structured metasurface at varying levels of magnification. The scale bars from left to right are 10 mm, 200 μm, 50 μm, and 500 nm [40]. Adapted with permission from [40]. Copyright 2020 American Chemical Society. (E) A dual-bias active metasurface LiDAR device. The unit cell structure is shown on the right [41]. Adapted by permission from [41]. Copyright 2021 Springer Nature. (F) Time-modulated metasurfaces (left) bias each antenna with a periodic signal (middle). Phase delay between antenna elements form a phase gradient proportional to sideband order (right) [42] Adapted from [42] / CC BY 4.0.

The pioneering work for tunable phase gradient metasurfaces was a study by Huang et al. [35] based on metal–insulator–metal (MIM) resonator antennas incorporating ITO as the tunable material. The metasurface used design principles similar to amplitude modulator metasurfaces [36], [37], [38]. For this structure, ITO and alumina were deposited on a gold back plate; a gold stripe antenna array was then deposited and patterned on top of the oxide thin films (Figure 2A). By constructing the metasurface in this way, each antenna supports a magnetic dipole resonance near the operational wavelength. Applying an electrical bias across the MIM capacitor structure induces charge carrier accumulation in a ∼1 nm layer on the boundary between the ITO and alumina, locally inducing a unity-order change in the material permittivity (Figure 2B). Huang et al. used this mechanism to shift the antenna’s resonance wavelength, measuring reflection phase modulation by each antenna of up to 180° with 2.5 V of applied bias. The beam steering functionality in this work was experimentally implemented by connecting alternating groups of antennas across the array. In this way, reflection intensity was switched between normal reflection and reflection into the −1st and 1st diffraction orders, at 76°, 40°, and 29° with 4-, 6-, and 8-antenna periodicity, respectively.

Shortly after, many theoretical studies were carried out exploring the use of TCO-incorporated resonant plasmonic antennas. Park et al. [43] investigated the operation of the same metasurface design principle at mid IR frequencies using coupled mode theory to model the behavior of reflection phase. The resonantly scattered light experiences a phase shift of up to 180° with voltage swept from −40 V to 40 V at 6 μm wavelength. Forouzmand et al. [44] simulated a TCO-tuned plasmonic antenna metasurfaces with a linear phase gradient rather than a step phase profile, putting forth the first proposal for a 2D TCO active metasurface. In the study, up to 30×30 arrays of gold square patch antennas were modeled with finite difference time domain (FDTD) simulations. A constant reflectivity of 4% was predicted, and the linear phase gradient was approximated by capping the ideal phase profile to 0°–250°. Later, the behavior of vertical and horizontal antenna stacks were simulated in dual frequency operation [45].

In addition to the original MIM antenna design, dielectric materials were also investigated an avenue to create resonant structures. Metal-oxide-semiconductor antennas could replace top gold electrodes with doped silicon to achieve a dielectric resonant structure [46]. Unlike previous structures, the unit cell geometry here was designed to support spectrally close electric and magnetic resonances. When bias is applied, the resonances merge to satisfy Kerker’s condition, and constant reflectivity of 16% was achieved while the reflected phase shifted 180°. However, the structure relied on an intricate multilayer ITO-alumina laminate for enhanced tunability, which would increase fabrication complexity and response time. A later theoretical work used a dielectric substrate (Figure 2C) to accommodate transmission operation [39]. Reflection and transmission were achieved using spectrally separate resonances individually accessible via transverse electric (TE)- and magnetic (TM)- polarized light.

Following these initial studies, Shirmanesh et al. [47, 40] proposed a fishbone antenna array operating in reflection mode, improving on Huang et al.’s work. The authors incorporated a layer of hafnium aluminum oxide laminate (HAOL), fabricated by alternating atomic layer deposition of hafnium oxide and aluminum oxide. This material simultaneously achieves high DC permittivity and high breakdown field, which increases the optical field’s overlap with the active material and enables higher applied voltages, respectively. The group also tried a dual gated arrangement which achieved a phase shift slightly higher than 300° [47]. By modulating the phase of each element in increments of 90°, the authors experimentally achieved the first demonstration of high-speed discrete beam steering in tunable metasurfaces (Figure 2D), where the use of highly conductive electrodes allowed for an angle to angle switching speed of up to 10 MHz. Despite this, the covariance of phase and amplitude introduced by the metallic loss resulted in diffraction orders with an achievable SMSR lower than 10 dB.

In 2021, Park et al. achieved full 360° phase modulation with constant amplitude by building on the dual gate structure, creating a working LiDAR device illustrated in Figure 2E [41]. A structure consisting of gold antennas patterned on a dielectric–ITO–dielectric–aluminum material stack was analyzed using temporal coupled mode theory. Here, carrier accumulation and depletion at the top and bottom ITO interfaces was controlled independently. At the same time, the coupled mode formalism revealed the resonator frequency and loss to be two independent parameters that control complex reflection. In previous studies, these parameters were covariant due to the use of single gate biasing. However, due to the structural asymmetry, the application of two independent gate voltages grants control over two degrees of freedom, allowing the authors to probe any reflection in the vicinity of the complex plane origin. Nevertheless, SMSR (2.7 dB) and field of view (7.7°) were severely limited in this device due to a large metasurface unit cell, and the diffraction efficiency was about 1%. However, the device showed decently high speed (170 kHz) thanks to the relatively high conductivity of gold and aluminum.

Alternatives to the transparent conducting oxide platform have also been explored for modulating resonant metasurface antennas. Phase change materials (PCMs) have long been part of the toolbox of solid-state optical active media, facilitating refractive index control through rapid and reversable switching between amorphous and crystalline phases through electrical, thermal, or optical means [48]. Nonvolatile PCMs such as GST (usually compounds with chemical formula Ge_*x*_Sb_*y*_Te_*z*_) alloys have enabled bistable operation for memory devices with nanosecond-scale switching time and typical lifetimes of billions of cycles [49]. New PCMs have been developed with optically important characteristics: the low absorption of GSST (Ge_2_Sb_2_Se_4_Te_1_), SbSe, and GeTe may spur developments in photonic applications [50, 51].

Yin et al. superimposed two plasmonic metasurfaces with different resonant wavelengths on a Ge_3_Sb_2_Te_6_ substrate for switching [52]. At 3.15 μm, GST’s amorphous phase activates one metasurface while its crystalline phase activates the other, deflecting circularly polarized beams in one of two discrete directions. Not limited to discrete material phase switching, GST and other PCMs have been shown to form intermediate states by the mechanism of partial nucleation [53, 54]. Cao et al. designed an array of four Au-GST-Au antennas for beam steering and simulated optical heating by femtosecond laser pulses [55]. Increasing antenna width over the array area created non-uniform heating, imparting a phase gradient that increased with pulse energy. However, collective phase changes such as this does not properly generate phase gradients in larger arrays with many 2*π* cycles.

For non-memory high speed switching applications, the volatile PCM VO_2_ is used to tune the resonance of plasmonic antennas [56]. Following the design of Huang and similar experiments [35, 43] to provide the experimental basis for phase gradient PCM metasurfaces, Kim et al. leveraged vanadium dioxide as the active material within plasmonic antennas to achieve reflected phase modulation [57]. By uniformly joule heating a patterned gold layer, the authors induced an amorphous-to-metallic phase transition in VO_2_ at *T*_c_ ∼ 340 K. This changes the permittivity from ∼−5 to 5 over its entire volume from ∼1500 nm to 1900 nm, with the effect being more pronounced at longer wavelengths, and induces up to 180° measured phase shift in each antenna. The effective permittivity of VO_2_ was modeled using the Bruggeman effective medium approximation as the volume fraction of each phase continuously changes. The phase change achieved here suggests the viability of a PCM phase gradient beam steering device with individually tuned antennas. However, due to the heat capacity and thermal diffusivity of the metasurface, only ∼2 Hz switching speed was measured (10 Hz for optical pulse heating) with significant hysteresis and amplitude modulation. The design of compact antennas with lower VO_2_ volume was suggested to improve switching time, as was experimentally demonstrated with a previous VO_2_/Au bowtie-based tunable hologram device [58].

Wu et al. exploited the quantum confined stark effect (QCSE) to achieve refractive index modulation, and therefore reflectance modulation, on a III–V semiconductor platform [59]. In this study, resonant antennas were patterned from a multiple quantum well stack; light leakage into the substrate was prevented by a distributed Bragg reflector mirror. To efficiently induce a spectral shift, a resonant mode was chosen such that its field profile overlaps with the multiple quantum wells. By applying an electric field across the MQW stack, the QCSE can facilitate a refractive index change of about Δn=0.01 . To perform initial testing, a thin film Fabry–Perot cavity was constructed, and applying 10 V shifted the resonance wavelength by 0.8 nm. The authors then fabricated the full metasurface and achieved a maximum of 70° of reflected phase shift by applying a voltage of 7 V. However, the reflected field experiences strong amplitude modulation of up to 250%. Discrete beam steering was further demonstrated by applying periodic step modulation. The device has a high theoretical tuning speed of 90 MHz due to the conductivity of doped III–V materials.

Electro-optical (EO) polymers are ultrafast Pockel’s effect materials whose tunability figure of merit (*r*_33_) and thermal stability have seen marked improvements in past years, with EO polymer Mach–Zehnder interferometer modulators achieving signal bandwidths of up to 500 Gbit/s [60, 61]. These materials can be easily incorporated through spin coating, and promising developments are observed in spatial light modulator metasurfaces [62, 63]. A study of amplitude modulation metasurfaces with the design language of previous TCO devices [36] gave inconclusive results possibly due to polymer degradation during deposition and patterning of gold contacts [64].

Among tunable materials, liquid crystals (LCs) have one of the highest tuning efficiencies but one of the slowest tuning speeds; nevertheless, we discuss a few notable works on this front. Komar et al. infiltrated liquid crystals into a linear phase gradient metasurface and thermally switched between its nematic and isotropic state to change metasurface element scattering characteristics [12]. Heating the LC by 60 K triggers the phase change and switches the transmitted beam between 0° and 12° deflection. Reconfigurable metasurfaces can be designed with individual unit cell control for better versatility. Li et al. fabricated an otherwise homogeneous metasurface with dynamically applicable phase gradient through rotating LC directors at different unit cells [11].

Throughout the development of reflection mode electrically tunable phase gradient metasurfaces, a persistent challenge has been to achieve a phase change of 360° with a constant amplitude profile. To date, resonant reflection phase tunability rarely exceeds 300°, thus clipping the optimal phase profile. One limitation is that the dielectric breakdown of the insulating and active materials precludes the use of larger modulation voltages. Additionally, the presence of absorbing materials such as ITO and metals severely diminishes reflection amplitude near resonance, causing varying amplitude, which is typically undesirable in conventional phase gradient metasurface design. To circumvent this limitation, several different avenues are being explored.

To address the problem of amplitude-phase covariation, Thureja et al. [65] used a genetic algorithm that considers reflected phase and amplitude simultaneously to optimize metasurface directivity. The directivity here is defined as the peak intensity divided by the average angular intensity. Borrowing Shirmanesh et al.’s [40] fishbone metasurface design, the author allowed the genetic algorithm to create a nonintuitive voltage profile and achieved a directivity of 72.7 and SMSR of 13.2 dB. The algorithm-optimized phase-amplitude distribution performs better than a simple linear phase gradient, which attains only directivity and SMSR of 39.5 and 6.8 dB, respectively. The author also explored limiting the tunable phase range of each metasurface element to 150°, 180°, 210°, and 240°, and a good directivity of above 60 could be achieved for a phase range as low as 210°. Intuitively, the optimization algorithms revealed a persistent tradeoff between directivity and efficiency, which is defined by total reflected power per constant input power. Whereas a high directivity can be achieved with low diffraction efficiency, incorporating diffraction efficiency into the algorithm’s objective causes the directivity to suffer.

To evade narrow band operation arising from the resonant metasurface platform, Salary et al. [66] explored the use of time modulated metasurfaces (TMMS). The physical principles of TMMSs are summarized as follows: light impinging on time modulated metasurface elements experiences frequency conversion into different harmonic orders, called sideband signals, spaced at integer multiples of the modulation frequency. The reflected sidebands undergo a phase shift proportional to the modulation phase delay and the harmonic order. In other words, a phase gradient in the modulation signals generates phase gradients in reflection at sideband frequencies. Additionally, modifying the metasurface modulation amplitude can independently modify the scattered field intensity in an arbitrary pattern. The initial physical proposal based on the concept of TMMSs was a structure consisting of graphene-wrapped doped silicon nanowires, operating at terahertz frequencies. Near-infrared operation using TCO metasurfaces was later theoretically studied [42]. In general, intensity of the scattered harmonics depends on the spectral composition of the periodic modulating waveform. Efficient conversion into a particular harmonic order can generally be achieved by optimizing the waveform with genetic algorithms, shown in Figure 2F [67].

Resonant metasurfaces modulated electrically using TCOs, such as ITO, are a promising platform to achieve arbitrary 1D beam shaping at high speed, limited only by the rise time induced by the capacitive effects of these devices. By adjusting the spectral features of each metasurface element through modifying material permittivity, researchers have achieved independent control over the phase and amplitude of scattered light. However, active phase gradient metasurfaces suffer from operation complexity associated with requiring hundreds of independent electrical channels to operate a few-hundred-micron device. These types of space and operational constraints are the primary reason two-dimensional beam steering and shaping have only been theoretically explored. Recently, time modulated metasurfaces have become an interesting avenue to further eliminate bandwidth and phase constraints. However, most active metasurfaces rely on external light sources, precluding the prospect of complete on-chip integration, and operate at low throughput efficiency due to resistive losses in the typically metallic platforms, which causes strong phase-amplitude covariation. This motivates the development of active metasurfaces with integrated emitters and the full exploitation of time modulation profile to access different harmonic emission sidebands.

## 4 Slow light beam scanning

For applications requiring only 1D beam steering, significant progress has been made using slow light to achieve enhanced tunability and field of view. Optical waveguides are characterized by their frequency dispersion relation, which determines the wave number of the optical mode and its group velocity. Group velocity is quantified by the first derivative of optical mode frequency with respect to wavenumber and is physically interpreted as the optical mode’s energy velocity, or the speed at which an optical pulse propagates. Waveguides supporting modes with reduced group velocity are called slow light waveguides, and naturally have enhanced light–matter interaction, allowing the wave vector of light to change drastically with respect to frequency. This can be understood graphically, as a small frequency shift in a waveguide mode creates a large momentum change. To out-couple light at a particular angle, one can either inject a slow light mode of varying wavelength or inject a mode at a particular wavelength and directly tune the waveguide dispersion through electro-optic or thermo-optic effects. In the past, various works have exploited the high sensitivity of the photon wavenumber with respect to refractive index perturbation to create compact sensors [68], [69], [70], switches, and modulators [71, 72]. This section of the review discusses the use of slow light engineered leaky waveguides for beam shaping.

**Figure 3:**
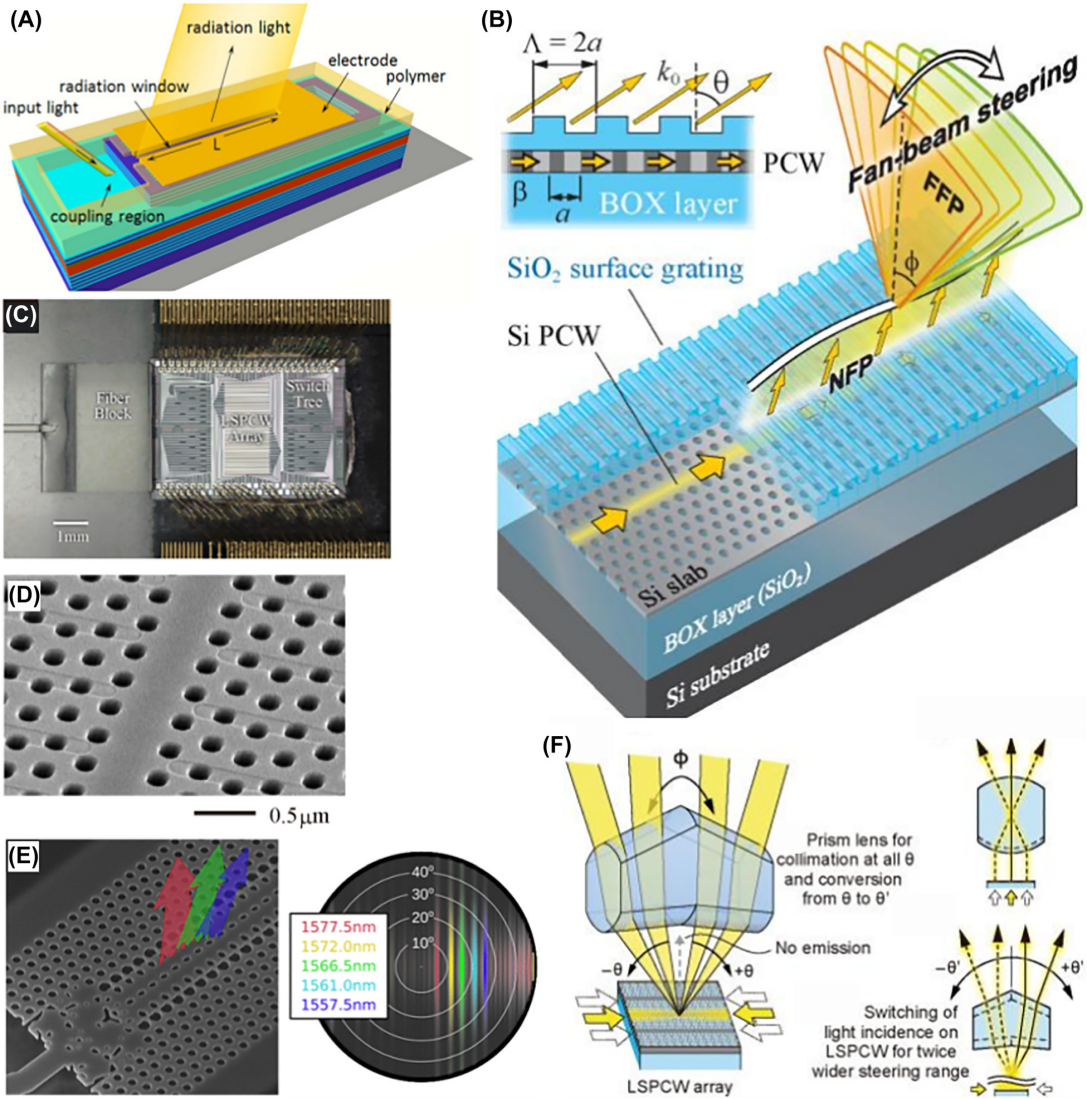
Slow light waveguide beam scanning. (A) Schematic of a VCSEL cavity slow light waveguide [23] Light is coupled in from a facet and is amplified along the cavity length. Adapted with permission from [23] © The Optical Society. (B) Doubly periodic shallow corrugations along a lattice-shifted photonic crystal waveguide (LSPCW) for coupling light into free space [76]. Adapted with permission from [76] © The Optical Society. (C) A fabricated LSPCW array, along with a switching structure [77]. (D) LSPCW with grating structure cut into the upper silica cladding [77]. The third row of holes is shifted slightly in the waveguide direction. Adapted from [77] / CC BY 4.0. (E) Inverse-designed photonic crystal slow light waveguide and coupler (left) with its far field pattern (right) [78]. Adapted with permission from [78]. © 2021 American Chemical Society. (F) Dual purpose lens for reducing the gap at normal emission (lower right) and collimating and redirecting transverse emission (upper right) [79].

The physical principles of slow light beam steering devices are exemplified in the early works of Koyama’s group [23]. The initially conceived device architecture resembles that of a horizontally elongated vertical-cavity surface emitting laser (VCSEL), as seen in Figure 3A. One end of the structure is milled to couple light from free space into the Bragg mirror cavity. The resulting waveguide mode is confined from the sides by total internal reflection and from the top and bottom through Bragg reflection. The top Bragg mirror is leaky, allowing laser light to escape. Using semi-analytical calculations, the emission angle can be adjusted over 70° by sweeping the input wavelength over a range of 40 nm around 900 nm. The VCSEL-derived architecture also allows the device to act as a laser amplifier so that any energy lost through radiation into the far field could be compensated by stimulated emission. In this way, the injected optical mode achieved a propagation distance of 1 mm, corresponding to a divergence angle of 0.025° and a far field resolution of *N* > 1000. Due to the transverse confinement of the optical mode, the far field for each leaky mode has a large angular spread in the transverse direction, resulting in a characteristic “fan beam”. These results were later verified experimentally [73], [74], [75].

Although VCSEL photonics can be used for wavelength modulated beam steering, the relatively large physical size of VCSEL waveguides imposes certain constraints. First, the slow light mode must be externally injected at an angle, requiring an extra milling step to create the coupling interface, and making device assembly tricky. More importantly, the speed at which the emission angle can be tuned is severely limited. The optical mode resides within thick Bragg mirrors that have significant heat capacity, reducing thermo-optic tuning speed [80]. To circumvent these problems, several authors have investigated integrated silicon photonics as an alternative means to achieve slow light, focusing on photonic crystal waveguides (PCW).

Photonic crystal defect waveguides (defect PCWs) are designed by creating line defects in photonic crystal slabs, which are periodic dielectric structures with sub-micron thickness. Light propagates through these defects, confined in the lateral directions by the patterned material’s photonic band gap and in the vertical direction by total internal reflection [81, 82]. These waveguides typically operate below the light cone, with the optical mode’s planar momentum exceeding that which is allowable by free space radiation.

In 2017, Kondo et al. initiated a line of research to create beam steering devices with slow light PCWs [76]. Embedding a photonic crystal waveguide patterned on 220 nm SOI (silicon on insulator) under a layer of silica, the author used a grating etched onto the top surface of the silica to perturb the waveguide mode and couple its energy into free space, illustrated schematically in Figure 3B. The grating period is selected to be double the photonic crystal period, folding the Brillouin zone in half to ensure radiation into a single direction. The waveguide dispersion was designed by starting with a so-called W1 defect waveguide, consisting of a row removed from a triangular lattice of holes, and uniformly shifting the holes of the third row on each side. The group index of this now lattice-shifted photonic crystal waveguide (LSPCW) was enhanced to around 20 over a 30 nm wavelength range around 1550 nm. The resulting field of view was 23°, corresponding to a sensitivity of ∼0.8°/nm. To characterize the propagation of the waveguide mode, the authors determined the propagation loss of the unperturbed waveguide to be about 20 dB/cm. For efficient optical throughput, the amount of useful radiative loss must surpass the unperturbed propagation loss, which arises from fabrication disorder. Otherwise, much of the optical power will be scattered and lost. The decay rate induced by the grating turns out to be about 50 dB/cm, indicating that useful radiative loss dominates. The mode intensity exponentially decays with a characteristic length of 620 μm, generating a corresponding theoretical beam width of 0.13°. However, measurement limits constrained the beam width to 0.23°, resulting in a resolution of ∼100. Naturally, large divergence was observed in the transverse direction due to mode confinement in that direction.

Following this initial work, Abe et al. improved on the design by employing a doubly periodic perturbation in the photonic crystal structure itself [83]. By changing the radius of every other hole in the propagation direction, the translational symmetry of the photonic crystal is naturally reduced, and the previously confined slow light waveguide mode is brought into the light cone. This introduces some robustness by avoiding the sensitive cladding thinning process required in Kondo et al.’s design. At a 10 nm difference in hole radius, the radiative propagation loss increased to a value of 150 dB/cm. Furthermore, quasi-2D beam steering was achieved by means of fabricating multiple such waveguides in parallel. A cylindrical lens above the array then collimated and directed the radiation from each waveguide in a particular direction, eliminating the wide ∼20° transverse beam divergence.

Exploiting the advantage of a small device volume, Takeuchi et al. explored use of the thermo-optic effect to directly tune the out-coupling angle [84]. Here, two methods of thermo-optic tuning were investigated. The first method directly applied Joule heating to the waveguide. The silicon was doped everywhere except the waveguide center, where it remained an intrinsic semiconductor. Applying a voltage across the waveguide resistively heated the center, changing its refractive index and therefore also its mode frequency. The second method was to place titanium nitride (TiN) heaters parallel to the waveguide and heat the waveguide through thermal conductivity. The first method proved to be more efficient and faster due to the local nature of heat injection. Using the so-called p–i–p doping pattern, 26° of steering was achieved using only 1.3 W of power, whereas devices controlled using TiN heating required 4.6 W. Additionally, the p–i–p waveguide’s low modulated volume allowed beam scanning at speeds up to 10 kHz, while TiN waveguides had a slower speed on the order of 1 Hz.

By design, directionally out coupled light is limited to either positive or negative angles, depending on the sign of group velocity. Previous works have coupled input light from both ends of the waveguide to double the resolution and FOV, but this arrangement makes the FOV discontinuous. Light out-coupling at zero wave vector is prohibited by zero group velocity, meaning that light does not propagate into the waveguide at all. To patch the discontinuity between positive and negative angles, Maeda et al. designed a lens-like optical element to remap the far field, refracting light from the two discontinuous angle ranges toward the normal direction, hence closing the small angle gap around normal emission (Figure 3F). Simultaneously, the author also collimated the dispersive transverse profile to improve directionality [85].

Other designs were studied, discovering new tradeoffs. Ito et al. used doubly periodic bulk photonic crystal mode, confined laterally by index guiding [86]. This distributed waveguide somewhat diminishes transverse dispersion by delocalizing the leaky mode. As a result, the group index was somewhat decreased, along with both tuning depth and sensitivity. A bulk mode also meant higher heat capacity and lower thermo-optic tuning speed. Tetsuya et al. stacked multiple waveguides with independent light sources end to end to circumvent mode decay and studied the stack’s beam shaping characteristics [87]. To take full advantage of this arrangement, phase shifters need to be implemented after splitting the power. Otherwise, diffraction effects prevent continuous tunability.

More recently, Ito et al. utilized a shallow grating cut directly into the photonic crystal surface to preferentially couple light in the upward direction, doubling the radiative efficiency [79]. Tamanuki et al. then fabricated an ensemble of these devices, together with the proper bulk optical elements, to produce a waveguide array capable of radiating into a 40° × 8.8° FOV, corresponding to over 10^4^ resolution points (Figure 3C, D) [77]. The device operated with fully electrical switching (between transverse angles) and scanning, and its total power consumption remained below 1 W. Vercruysse et al. used standard photonic inverse design to create low loss couplers and mode converters for slow light beam steering applications, as shown in Figure 3E [78]. The waveguide supercell could be freely tailored to have positive or negative dispersion. Furthermore, the group index could be tuned to be large and constant over the operational frequencies. Finally, these optimizations were shown to be possible for both even and odd waveguide modes.

For one dimensional wavelength independent beam steering, slow light photonic crystal waveguides provide a compact and high speed nanophotonic platform, capable of achieving a large field of view and resolution. Thermo-optic tuning provides an effective means to induce a refractive index change in the waveguiding material, allowing for constant-wavelength operation at speeds of up to tens of kHz. Compared to active resonant metasurfaces, slow light waveguides hold a significant advantage in their operation simplicity. These devices can be further exploited for 2D beam steering, with a single pair of electrical contacts needed for each desired angle in the transverse direction.

## 5 Optical phased arrays

Optical phased arrays (OPA) are a quickly maturing technology for producing directional beams. The general operation of these devices is as follows. Light is coupled into an integrated waveguide bus, and some waveguide splitter routes energy equally into many optical waveguides. The device architecture can then be engineered to produce a phase gradient across the waveguide array, and the phase shifted waveguide modes are coupled into free space by a grating structure. Over the last decade, an immense amount of research has been conducted on variations of this photonic architecture to achieve larger angle range and better far field quality.

**Figure 4:**
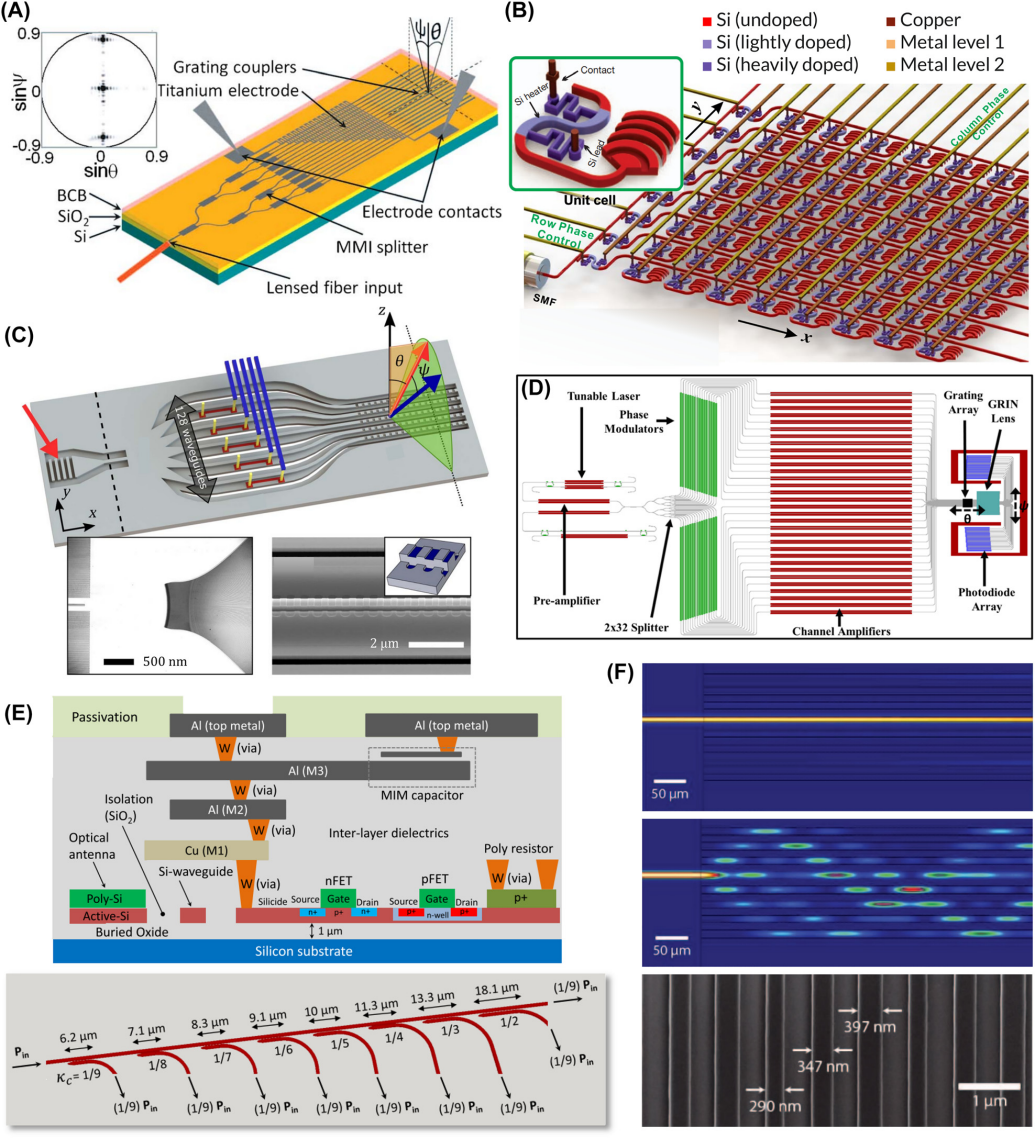
Optical phased arrays. (A) An optical phased array using titanium heaters with length linearly increasing across the array [88]. Adapted with permission from [88] © The Optical Society. (B) An 8 × 8 OPA fed from a single optical fiber [89]. The design of a single antenna is inset, showing an S-bend phase shifting section for high-speed thermo-optic phase tuning. Adapted with permission from [89] © The Optical Society. (C) An array of 128 individually tuned waveguides with power input from a star coupler [90]. Adapted with permission from [90] © The Optical Society. (D) OPA fabricated on a hybrid III–V/silicon platform with integrated light source and photodiode array for feedback [91]. Optical gain elements are indicated in red. Adapted with permission from [91] © The Optical Society. (E) SOI CMOS architecture for a scalable optical phased array (top) [92]. The elements of each row couple power evenly from a single bus waveguide (bottom). Adapted by permission from [92]. © 20XX IEEE. (F) Varying waveguide width reduces crosstalk (top) from waveguide coupling (middle) [93]. Fabricated structures are shown in the bottom figure. Adapted with permission from the authors.

The general operation of this class of devices can be introduced with a seminal work by Acoleyen et al., in which a simple yet capable beam steering device was constructed and fabricated on SOI [88] (Figure 4A). Here, NIR laser light was coupled into a waveguide via a lensed fiber. Through a tree of multimode interferometers (MMIs), energy was transferred evenly over an array of 16 waveguides, each spaced 2 μm apart. Each waveguide mode passed through TiN thermo-optic phase modulators with length linearly increasing across the array, producing a linear phase gradient. A grating with a subwavelength period of 630 nm is etched into each waveguide, coupling light into free space.

Such a simple device is already capable of 2D beam steering. By sweeping the wavelength between 1500 nm and 1600 nm, the angle of the beam along the direction of the waveguides (i.e., the longitudinal direction) can be swept over a range of 14.1°. From a decay length of 35 grating periods, a beam width of around 2.5° can be achieved. For tuning the angle in the direction perpendicular to the waveguides (i.e., the phased array direction or the transverse direction), a current was run through the titanium heating elements, shifting the waveguide refractive index by exploiting silicon’s relatively large thermo-optic coefficient of 1.86 × 10^−4^ K^−1^ in the NIR [94]. In this device, applying voltage bias creates 2.3° of transverse angle change. The total field of view for this device is then 2.3° × 14.1° with a beam width of 2.7° × 2.5°. Henceforth in this review, we report the field of view and beam width with the transverse values first.

A common theme across many studies is the battle against energy loss into side lobes. Acoleyen et al.’s 2 μm period between waveguides led to diffracted beams appearing at large angles near the edge of the far field, and early research works focused heavily on this problem. Xiao et al. conducted a theoretical study based on a wavelength-controlled beam steering device [95]. An array of waveguides takes their input from a spatially coherent source. By modifying each waveguide so that the path length linearly increases across the array, the light at the end of each waveguide forms a directional beam. The effective index, and therefore path length, varies as wavelength is changed, and different wavelengths are projected in different directions.

However, a linear waveguide array with waveguide spacing greater than half the free space wavelength will necessarily couple light into multiple diffraction orders. Xiao et al. employed an irregular array, with random waveguide spacing to suppress the side modes. Similarly, Hosseini et al. proposed a 3D structure consisting of a 2D array of silicon waveguides [96], similar to stacking many 1D waveguide arrays vertically. The authors proposed individually tuning these waveguides with thermo-optic phase shifters. In this way, a fully 2D phase gradient can be achieved, which projects a beam at an arbitrary angle in the far field. To suppress diffraction, the group proposed constructing each dimension of the array by combining multiple subarrays, each of different periodicity. In this way, diffracted energy is spread over many angles, allowing a steering half angle of 45° in both directions. To experimentally verify this design principle, Kwong et al. fabricated on SOI an irregular optical phased array with 12 waveguides [97]. A lensed fiber provides power to 12 waveguide modes, whose phases were independently set by 12 phase modulators. In this case, the waveguides are terminated by a silicon slab and act as point sources in a 2D plane.

At around the same time, other developments have continued to improve the performance parameters of OPAs. Doylend et al. increased the maximum steering angle of Acoleyen’s device by adding independent phase shifters for each waveguide [98]. Because long silicon waveguides accumulate significant phase errors, the group employed an algorithm to fine-tune the output phases, optimizing for good main lobe quality and high SMSR (>10 dB) within the first diffraction order. The use of a regular array here introduced diffraction, limiting the tunable angle range down to 20° × 14°. The beam width was 1.6° × 0.6°, corresponding to a total resolution of ∼13 × 23.

Wavelength tuning simplifies integrated nanophotonic phased array design and is made possible by grating-like antenna arrays. Nevertheless, single wavelength operation of a phased array is highly desirable for many applications. Sun et al. fabricated an 8-by-8-antenna 2D phased array, experimentally demonstrating 2D beam steering at a single wavelength for the first time [89]. From a fiber coupled waveguide, energy is distributed over a row of eight waveguides, then over 8 antennas on each row (Figure 4B). The phase modulator for each antenna is designed to be compact, fitting within the space of a single pixel and taking the shape of two waveguide half bends. By introducing waveguide bends, the waveguide mode is moved away from the doped silicon contacts through which current is injected for resistive heating. Electrical contacts are connected over each column and row for only 2*N* electrical channels for an *N* × *N* array.

The propagation length of the radiating waveguide modes of optical phased arrays is an important consideration due to its impact on the far field beam width. Kwong et al. narrowed the beam width in the longitudinal direction of an OPA by carefully controlling the grating perturbation [99]. Instead of directly etching the waveguide array, which requires fine control due to silicon’s high refractive index, the waveguide grating was etched out of deposited amorphous silicon, separated from the waveguides by a layer of low index silica. This way, the periodic perturbation is applied only on evanescent tails of each waveguide mode. These improvements resulted in a 20° × 15° FOV and a 1.2° × 0.5° beam width.

So far, waveguide arrays have only been individually modulated, but other modulation schemes are also possible. Yaacobi et al. created a cascaded phase modulator array for fast beam scanning across the designed field of view [100]. Instead of splitting a single waveguide through cascaded MMIs, energy was coupled sequentially from the main waveguide. A double bend modulator as found in Sun et al. accumulates some constant phase shift after every coupler. This device has the advantage of being easy to operate, with a single electrical signal inducing the transverse angle change. However, this design precludes the removal of phase errors accumulated from fabrication imperfections. At the same time, the device geometry allows for a steering range up to 51° and a beam width of 3.3° in the phased array direction. The small modulator volume allows speeds of up to 100 kHz.

Tightly integrated photonic platforms provide various advantages towards compact array design. Despite the optimization strategies to improve thermo-optic phase tuning speed, other platforms may provide easier means of fast optical modulation. Aflatouni et al. explored near-infrared image projection and beam generation through the electro-optic effect in silicon [101]. Conventional p-i-n modulators capable of 200 MHz fed a 4 × 4 array of optical antennas with 50 μm pitch. Phase-amplitude covariation caused by material absorption was present but manageable via an optimization algorithm. Abediasl and Hashemi fabricated a monolithic optical phased array transceiver using a CMOS SOI process [102], implementing thermo-optic amplitude and phase control locally around the each of the 8 × 8 antennas. Power monitors, the receive/transmit switch and calibration grating couplers were fabricated for device calibration. Like the antenna array of Sun et al., large array periods due to the integration of optical and electrical components (50 μm in [101] and 33 μm in [102]) reduce the achievable FOV and increase the beam width. Nevertheless, these devices pave the design language of tightly integrated optical phased arrays and in the future may be scaled for resolution improvements.

An important consideration for the commercialization of OPA technologies is light source integration. However, the lack of gain in silicon prevents this within a single SOI chip. Thus, SOI OPAs commonly rely on external light sources, which adds sensitive assembly steps and therefore more cost to the final device. A better way is to integrate lasers, amplifiers, and feedback elements all within a single chip. To demonstrate this concept, Hulme et al. fabricated a wavelength tunable OPA on a hybrid III-V/silicon platform, complete with a tunable laser source, channel amplifiers, and photodiodes (Figure 4D) [91]. In particular, laser light was provided by tunable Vernier ring lasers. To increase the emitted light intensity, amplifiers were placed before the waveguide splitters and after each of the 32 phase modulators. An array of photodiodes is situated posterior to the phased array grating to monitor unwanted phase errors caused by thermal changes and fabrication. The authors achieved a 23° × 3.6° FOV and a 1° × 0.6° beam width.

Pushing for higher FOV and resolution, Hutchison et al. fabricated a non-uniform phased array (Figure 4C) with a record high 128 waveguides, suppressing the beam width to 0.14° [90]. The same level of beam divergence is also achieved in the wavelength tuned direction, due to the weakly perturbative silicon grating. By implementing their non-uniform phased array design, the authors expanded the lateral FOV to 80°. Additionally sweeping the full wavelength range of over 100 nm results in a 17° longitudinal angle change. Over both axes, over 60,000 resolution points were achieved.

Although laser displays have been frequently mentioned as a potential use of OPAs, OPAs have largely only been designed for NIR operation due to the absorption of silicon at shorter wavelengths and because most LiDAR systems use invisible, eye-safe wavelengths. Silicon nitride (SiN) however is completely transparent at visible wavelengths, and SiN waveguides are standard in integrated photonics. Poulton et al. demonstrated SiN OPA operating at both NIR and 635 nm with record low beam divergence [103]. For NIR laser light, the author demonstrated a 1024-antenna array spaced at 4 μm to form a 4 mm by 4 mm array with 0.021° beam divergence. At 635 nm, the same number of antennas spaced at 2 μm forms a 0.5 nm × 0.5 nm aperture, creating a 0.064° × 0.074° beam. Unlike silicon, SiN can support very high powers, a virtue of its wide band gap and lack of two photon absorption. Additionally, its low index contrast with usual substrate and cladding materials reduces the severity of any phase error arising from fabrication imperfections.

To circumvent aliasing, non-uniform waveguide arrays are typically used, and general guidelines have been established in various studies, such as Komljenovic et al., which discussed the merits of different arrangements [104]. Alternatively, one can attempt to reduce the waveguide array spacing, at the risk of increased crosstalk. However, Phare et al. [93] leveraged a waveguide superlattice technique [105] to bring waveguides closer than ever before while maintaining low crosstalk (Figure 4F). By assigning waveguides different widths, the author created effective index mismatches between adjacent waveguides, reducing their mode coupling. In this way, the array pitch can be reduced to half of free space wavelength, guaranteeing single-lobe emission.

Recent research has included many efforts to accomplish single wavelength 2D beam steering. The most formidable challenge is to reduce the complexity, such that an *N* × *N* array of emitters (or equivalent) does not require *N* ^2^ electrical controls. Chung et al. explored a scalable architecture to limit the required number of digital to analog converters from *N* ^2^ to *N* on an SOI CMOS platform (Figure 4E) [92]. Through row-column phase addressing, 1192 optical variable phase shifters, and 168 optical variable attenuators can drive an array of 1024^2^ antennas.

**Figure 5:**
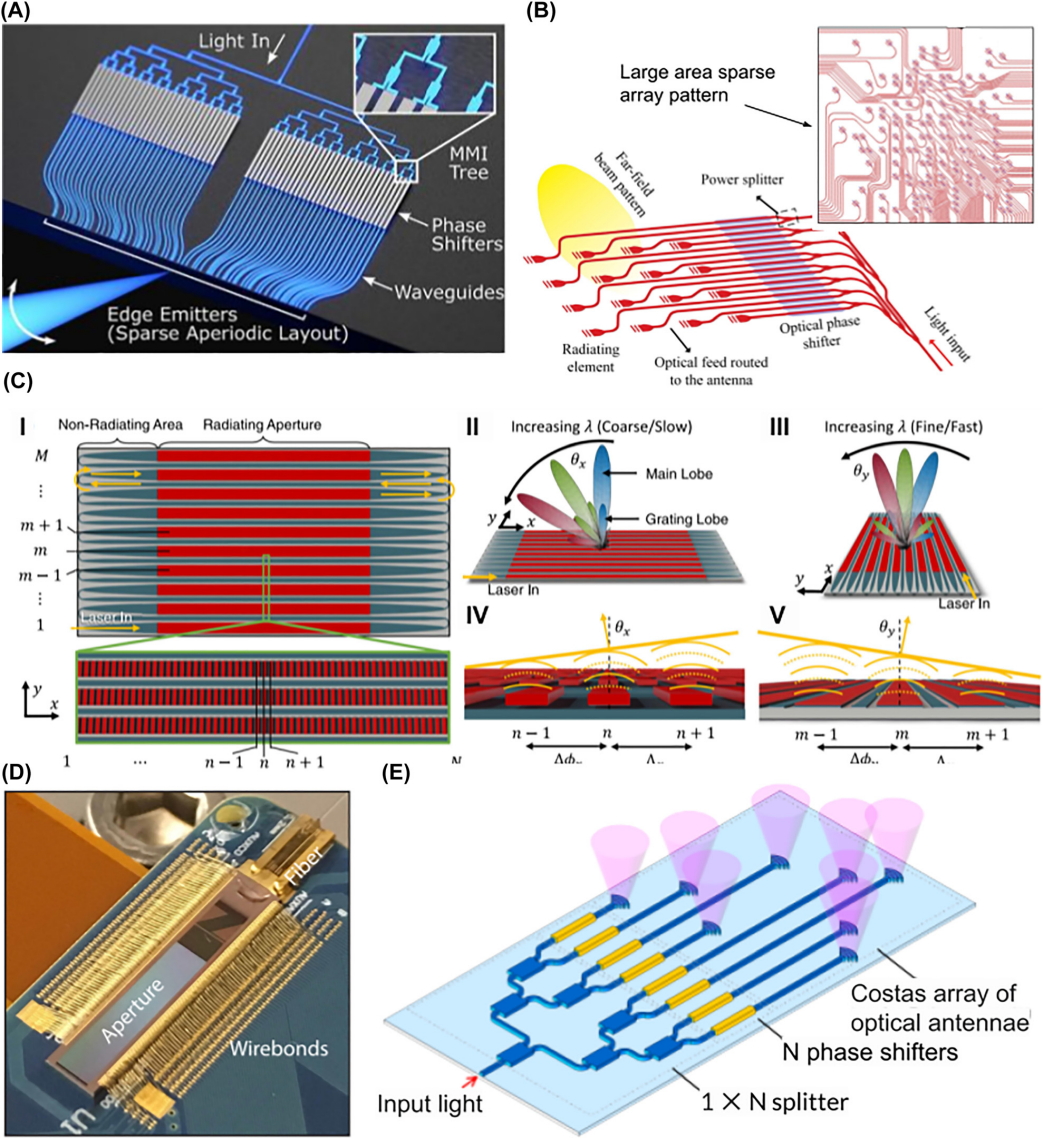
High performance optical phased array beam steering. (A) Blue light edge emitting optical phased array, with aperiodic antenna spacing [108]. Adapted with permission from [108] © The Optical Society. (B) A large area non-uniform antenna array [107]. The top right inset shows its sparse antenna distribution. Adapted by permission from [107]. © 2019 IEEE. (C) Serpentine optical phased array capable of 2D angle tuning by controlling wavelength only [109]. Light coupled from the bottom right is radiated travelling rightwards and returns to the next row, as shown in the device layout (I). Phase accumulation is much lower across each antenna (II) than across each row (III), producing slowly (quickly) varying phase gradients in the waveguide (transverse) direction (IV), (V). (D) A several-mm-large optical phased array for use as a LiDAR transceiver [110]. Reprinted with permission from [110]. © 2019 IEEE. (E) A Costas antenna array reduces the number of phase shifters required for single-wavelength angle tuning from *N* ^2^ to *N* [21].

Independently tuning the amplitudes and phases of 2*N* SOI waveguides, each functioning as the input for a column or row of an array, Ashtiani et al. uses interference between the column and row waveguides at each emitter to achieve 2D phase control within an 8 × 8 antenna array [106]. By the nature of this kind of modulation, the amplitude of each of the *N* ^2^ emitters is covariant with its selected phase, which is somewhat undesirable. The linear array design allows a 7° FOV, and the entire array spans 77 μm × 77 μm. Fatemi et al. explored a similar structure (Figure 5B), choosing to reduce complexity by only sparsely populating the emitter array [107]. On an SOI CMOS platform, standard optical phased array architecture was built apart from the waveguide antennas, which emit like those in the work of Sun et al. As the aperture size increased, antennas were placed more sparsely to compensate for the increased waveguide density. Although the SMSR achieved was not as large as that of a densely populated array, the SMSR was shown to be maintained above 19 dB despite the nonuniform emitter arrangement.

Another method to reduce control complexity even further is to replace the wavelength tunability with a large area thermo-optic phase modulator, as was done by Kim et al. [111]. Instead of sweeping wavelength, the effective index at the grating section of a traditional OPA may also be modulated using the thermo-optic effect. A 16-element array, using p-i-n heaters for individual elements and n-i-n heaters for the grating section, manages to sweep a 45.4° × 10° FOV with 3.2° × 5.8° beam width at 1550 nm. Alternatively, Tyler et al.’s implementation switches between OPAs designed for different transverse angles while the longitudinal angle is thermo-optically tuned, achieving 17.6° × 3° with beam width 4.3° × 0.7° [112].

Other optimizations have been made to improve the general function of OPAs. Common to most OPAs is some optical loss through the device substrate. Zhang et al. circumvented this by fabricating a typical beam steering device with a distributed Bragg reflector to prevent bottom-side leakage [113]. To reduce the necessary voltage needed for phase modulation, Miller et al. fabricated a multipass phase shifter by repeatedly converting and redirecting waveguide modes back through a heated waveguide section [114]. A 70° × 6° FOV was achieved while modulation power was reduced by an order of magnitude from standard values.

More exciting still has been the development of simpler and more elegant beam steering techniques. Serpentine OPAs have recently been demonstrated by Dostart et al. [109]. Here, a single degree of freedom, the wavelength, dictates the 2D emission angle in the far field (Figure 5C). In the longitudinal direction, waveguide dispersion dictates the angle, as usual. In the transverse direction, however, each waveguide is connected to the end of the previous through a flyback waveguide. The phase accumulated through forward and backward propagation forms the phase gradient needed for steering. Thus, the transverse angle sweeps quickly and repeatedly over its allowed FOV, limited by diffraction due to large waveguide spacing, while the longitudinal angle varies more slowly. A 1450–1650 nm wavelength sweep produces 16,500 addressable spots in a 27 × 610 array.

Very recently, a minimally populated antenna array has been proposed and fabricated, requiring only *N* phase shifters for *N* × *N* array resolution [21]. To accomplish this, the antennas were arranged in Costas arrays (Figure 5E), which are the 2D generalizations of Golomb rulers and have a delta-function-like autocorrelation function [115]. This guarantees maximum destructive interference of side lobes, which makes the rest of the far field accessible with a vastly reduced antenna count.

Large-area devices have been fabricated and operated for proof-of-concepts of practical applications. Poulton et al. used separate SOI OPAs as LiDAR transmitter and receivers on the same chip. The same group later also demonstrated data transmission over free space between two OPAs (Figure 5D) [110, 116]. At the same time, this technology is maturing into marketable form using CMOS SOI, moving computation and self-error correction onto the same chip [117]. For display technologies, miniaturization continues to bring NIR technology into the realm of visible light with a blue light tunable OPA making all the visible spectrum accessible (Figure 5A) [108].

Lastly, we briefly address notable works on conventional active platforms such as VCSELs and MEMS. VCSELs are monolithic lasers that achieve circular beam profiles and have been extensively commercialized for optical communication applications. VCSEL arrays routinely achieve periods of a few wavelengths, on par with most 2D non-grating nanophotonic phased arrays, whose large optical routing footprints restrict their minimum antenna spacing. Pan et al. phased the output of 4 × 4 square and hexagonal coherently coupled VCSEL arrays with liquid crystals [118]. Row and column biasing at up to 1 V induced ±1 ° angle change over a 5 ° FOV with ∼1 ° beam width. A larger scale study with tandem injection-locked VCSEL arrays with 50 nonuniformly distributed antennas demonstrated extended FOV of 2.2° × 1.2° with 7.7 dB SMSR and ∼0.31° beam width [119].

Despite the nonmechanical nature of most nanophotonic beam steering techniques, the low inertia of nanoscale MEMS systems allows them to be tuned reasonably fast. Yoo et al. fabricated an 8×8 array of microelectromechanically-actuated high-contrast grating (HCG) mirrors to create variable phase delay [120]. The design enabled 775 nm/2 vertical displacement with 18 V for *π* phase shift at 1550 nm. Designing for operation near mechanical resonance minimizes the device’s response time to 3.18 μs (5.83 μs) off to on (on to off). The same group also optimized the device operation through input shaping of the voltage signal to compensate for mechanical oscillations and closed loop feedback of device output [121].

Extending these works, Yang et al. created resonant antennas by placing HCGs above Bragg mirrors, where the resonant wavelength can be tuned through the cavity length [122]. This type of modulation along with the ability to tune quality factor through HCG design reduces the necessary MEMS actuation displacement and voltage, granting 1.7*π* ∼ 305 ° phase change by sweeping over 50 nm and 1 V, respectively. Owing to the large antenna structure size and the circuitry between individual antennas, both arrays have a pitch of around 35 μm, limiting FOV to some 2.5° at 1550 nm before magnification. The non-mirror area also increases unwanted specular reflection from the substrate. The FWHM of the second array was measured as to be around 0.3°, giving a resolution of ∼64.

## 6 Summary and outlook

The past few years have witnessed significant progress in the ability to generate directed free space radiation at arbitrary angles. Tunable resonant metasurfaces have been demonstrated to grant powerful control over spatial modulation of phase and amplitude. Plasmonic and doped semiconducting material platforms for constructing these metasurfaces hold the promise of ultra-high-speed modulation of angle and polarization. Just as exciting are the new and arguably more natural controls over amplitude and phase through time modulated metasurfaces. For one dimensional beam steering, the light matter interaction enhancement provided by slow light waveguides offers an elegant approach to couple light into free space with opportunity for CMOS integration. Integrated optical phased arrays provide a powerful way to modulate the angle of light in both angular directions in the far field, with much progress in achieving narrow beam width, large FOV, and high SMSR through clever array designs. To summarize the most relevant progress in the broad field of nanophotonic beam steering, we tabulate performance metrics of several notable studies. In the following few paragraphs and utilizing Table 1, we discuss the general challenges of constructing nanophotonic beam steering devices.

**Table 1:** Comparison of notable beam steering works from each device architecture. FOV, resolution, and beam width are reported as a single number for 1D steering. All values are experimental except those in parentheses, which are derived. Abbreviations: Input λ: input wavelength tuning; EO: electro-optic effect; QCSE: quantum-confined Stark effect.

	Ref.	FOV [°]	Resolution	Beam width [°]	Tuning mechanism(s)	Speed [MHz]	Power* [mW]	SMSR [dB]
Active metasurfaces	[35]	40	–	(2)	ITO carrier injection	>10	(700)*	–
	[40]	44×0	9×1	(2×2)	ITO carrier injection	–	–	3
	[59]	12	–	(0.8)	III–V QCSE	1	(0.016)*	–
	[41]	6	31	0.2	ITO carrier injection	5.4	(96)*	2.7
Slow light scanning	[23]	70	>1000	0.025	Input λ	–	–	–
	[76]	23	100	0.23	Input λ	–	–	–
	[84]	21	120	0.2	Thermo-optic	0.1	1300	–
	[79]	40×4.4	266×40	0.15×0.15	Input λ	−	−	−
Optical phased arrays	[91]	23×3.6	(23×6)	1×0.6	Thermo-optic	0.3	160	5.5
	[90]	80×17	570×120	0.140×0.142	Thermo-optic, input λ	–	–	8.9
	[107]	16×16	(20×20)	0.8×0.8	Thermo-optic	0.019	10.6	12
	[110]	56×15	(1400×375)	0.04×0.04	Silicon EO**, input λ	0.033	0.002	12
	[109]	70×6	(470×75)	0.15×0.08	Thermo-optic, input λ	0.15	1.7	7.5

*For active metasurfaces, values describe power consumption of *entire* metasurface at highest operating speed. For other devices, this is the power consumption *per antenna*. **Low speed due to control electronics.

Antenna spacing significantly influences far field quality through unwanted diffracted beams but are in principle less problematic for grating-like devices and metasurfaces. The grating-like devices (Figure 1B) of Sections 4 (Slow light beam scanning) and Section 5 (Optical phased arrays) circumvent side-lobe formation in the waveguide direction since each grating element, usually spaced <λ/2 , acts as an antenna. On the other hand, active metasurfaces couple incident radiation into tightly spaced MIM resonant antennas that do not inter-couple owing to subwavelength plasmonic optical mode confinement. In principle, diffraction-free near-180° steering should be possible, but the blessing of tight antenna integration is often diminished by phase-amplitude covariation in antenna reflectivity which introduces spurious diffraction lobes into the far-field. Table 1 illustrates the resulting severe reduction in SMSR compared to other devices.

All optical phased array devices of Section 5 (Optical phased arrays) require optical routing via waveguide networks, which is problematic due to waveguide coupling and crosstalk at small antenna spacing. An analysis by Zhang et al. deemed that, provided coupling is minimized through optimizing waveguide dimensions, the waveguide spacing can be tightened to ∼1 μm while suppressing κ to $\kappa^{-1}∼L_{\text{waveguide}}$ for Si waveguides embedded in SiO_2_ [113]. However, transparent materials like silicon nitride, useful in visible wavelength and higher power applications, have a lower refractive index contrast, decreasing optical mode confinement and increasing inter-waveguide coupling. The larger waveguide spacing required to avoid waveguide crosstalk reduces usable FOV for beam steering. As a result, nonuniform antenna layouts have received much interest as a way to disperse the optical power of diffraction lobes across the far field. In practice, spurious lobes may be suppressed to around 10 dB, close to the typical side lobe levels in uniform arrays that are induced by random phase errors.

Achievable device speeds depend critically on tuning mechanism and material, made obvious in Table 1. Refractive index changes in ITO-based metasurfaces are generated by carrier injection and therefore limited only by electronic transport properties. In practice, time delay is dominated by the antennas’ RC time constant, approximately $RC=L^{2}\epsilon_{0}\epsilon_{r}/\left(\sigma t_{a}t_{c}\right)$ . $L,\epsilon_{0},\epsilon_{r},\sigma ,t_{a},\text{ and }t_{c}$ are the antenna length, free space permittivity, gap dielectric constant, electrode conductivity, antenna thickness, and gap thickness, respectively. A square ${\left(40\;\mu \text{m}\right)}^{2}$ array of 50 nm thick gold antennas sandwiching 20 nm ITO and 5 nm alumina have response time on the order of 10 ps, corresponding to some 100 GHz. Switching energy is approximately the stored capacitive energy $∼CV^{2}=V^{2}L^{2}\epsilon_{0}\epsilon_{r}/t_{c}∼0.1\text{ nJ}$ . Power is fundamentally limited to ∼10 W, independent of device area. Contrast this to the power-speed relation of thermo-optic phase shifters, overwhelmingly used in non-metasurface devices. The observed trend in modulator designs points to a log–log relation between power consumption *P* and modulation bandwidth f : ${\log\;}_{10}\left(P/\left[\text{mW}\right]\right)∼{\log\;}_{10}\left(f/\left[\text{kHz}\right]\right)\times 2/3$ [24]. Extrapolating to even 10 MHz predicts waste heat on the order of 1 W per waveguide, suggesting a significant and perhaps fundamental roadblock in achieving high speed thermo optic beam steering.

Pushing for high performance beam steering beyond the state of the art may require carefully combining breakthroughs from disparate fields. For example, integrated waveguide driven metasurfaces may allow metasurface-level speed and directivity on an integrated photonics platform, and the subject of time-modulated metasurfaces seem ripe for tunable beam deflector experiments in the near-infrared. To achieve fully 2D beam steering, active metasurface engineers must contend with the challenge of compact integration to achieve full addressability on a 2D grid of pixels beyond individual row control. Row- and column-wise biasing such as that found in commercial display technology may provide a viable strategy. Further, emerging nanophotonic techniques offer great prospects for innovative designs. In particular, sparsely populated emitter arrays stand to benefit from emerging inverse design methods [78, 123], providing more powerful ways to tailor the far field. Phase modulation through nonlinear polymers may be an avenue to create extremely fast and power-efficient devices while keeping fabrication simple [124, 125]. Further, the rise of nanoscale 3D printing suggests more flexible ways of designing metasurfaces and waveguide arrays [126, 127]. Considering these developments, we believe nanophotonic phased array technology to be a versatile way to steer light at optical frequencies and are excited to see the development of even simpler and more capable photonic architectures.

## References

[1] de Galarreta C. R., Alexeev A., Au Y.‐Y. (2018). Nonvolatile reconfigurable phase-change metadevices for beam steering in the near infrared. *Adv. Funct. Mater.*.

[2] Tholl H. D. (2006). Novel laser beam steering techniques. *Technologies for Optical Countermeasures III*.

[3] Kim I., Martins R. J., Jang J. (2021). Nanophotonics for light detection and ranging technology. *Nat. Nanotechnol.*.

[4] Krishnan S., Crosby C. J., Nandigam V. (2011). OpenTopography: a services oriented architecture for community access to LIDAR topography. *Proceedings of the 2nd International Conference on Computing for Geospatial Research & Applications, New York, NY, USA*.

[5] Prakash D. S., Ballard L. E., Hauck J. V. (2021). United States Patent: 11151235-Biometric authentication techniques.

[6] Kedar D., Arnon S. (2004). Urban optical wireless communication networks: the main challenges and possible solutions. *IEEE Commun. Mag.*.

[7] McManamon P. F., Bos P. J., Escuti M. J. (2009). A review of phased array steering for narrow-band electrooptical systems. *Proc. IEEE*.

[8] Monk D. W., Gale R. O. (1995). The digital micromirror device for projection display. *Microelectron. Eng.*.

[9] McManamon P. F., Ataei A. (2019). Progress and opportunities in optical beam steering. *Quantum Sensing and Nano Electronics and Photonics XVI*.

[10] He Z., Gou F., Chen R., Yin K., Zhan T., Wu S.-T. (2019). Liquid crystal beam steering devices: principles, recent advances, and future developments. Crystals.

[11] Li S.-Q., Xu X., Veetil R. M., Valuckas V., Paniagua-Domínguez R., Kuznetsov A. I. (2019). Phase-only transmissive spatial light modulator based on tunable dielectric metasurface. *Science*.

[12] Komar A., Paniagua-Domínguez R., Miroshnichenko A. E. (2018). Dynamic beam switching by liquid crystal tunable dielectric metasurfaces. *ACS Photonics*.

[13] Hirabayashi K., Kurokawa T. (1993). Liquid crystal devices for optical communication and information processing systems. *Liq. Cryst.*.

[14] Smith N. R., Abeysinghe D. C., Haus J. W., Heikenfeld J. (2006). Agile wide-angle beam steering with electrowetting microprisms. *Opt. Express*.

[15] Zohrabi M., Lim W. Y., Cormack R. H., Supekar O. D., Bright V. M., Gopinath J. T. (2019). Lidar system with nonmechanical electrowetting-based wide-angle beam steering. *Opt. Express*.

[16] Römer G. R. B. E., Bechtold P. (2014). Electro-optic and acousto-optic laser beam scanners. *Phys. Procedia*.

[17] Nakamura K., Miyazu J., Sasaura M., Fujiura K. (2006). Wide-angle, low-voltage electro-optic beam deflection based on space-charge-controlled mode of electrical conduction in KTa_1−*x*
_Nb_
*x*
_O_3_. *Appl. Phys. Lett.*.

[18] So S., Park N., Lee H. J., Rho J. (2020). New trends in nanophotonics. *Nanophotonics*.

[19] Heck M. J. R. (2017). Highly integrated optical phased arrays: photonic integrated circuits for optical beam shaping and beam steering. *Nanophotonics*.

[20] Balanis C. A. (2016). *Antenna Theory: Analysis and Design*.

[21] Fukui T., Tanomura R., Komatsu K. (2021). Non-redundant optical phased array. *Optica*.

[22] Stutzman W. L., Thiele G. A. (2012). *Antenna Theory and Design*.

[23] Gu X., Shimada T., Koyama F. (2011). Giant and high-resolution beam steering using slow-light waveguide amplifier. *Opt. Express*.

[24] Chung S., Nakai M., Hashemi H. (2019). Low-power thermo-optic silicon modulator for large-scale photonic integrated systems. *Opt. Express*.

[25] Sun J., Kumar R., Sakib M., Driscoll J. B., Jayatilleka H., Rong H. (2019). A 128 Gb/s PAM4 silicon microring modulator with integrated thermo-optic resonance tuning. *J. Lightwave Technol.*.

[26] Sinatkas G., Pitilakis A., Zografopoulos D. C., Beccherelli R., Kriezis E. E. (2017). Transparent conducting oxide electro-optic modulators on silicon platforms: a comprehensive study based on the drift-diffusion semiconductor model. *J. Appl. Phys.*.

[27] Warren M. E. (2019). Automotive LIDAR technology. *2019 Symposium on VLSI Circuits*.

[28] Mardirosian R. (2018). LiDAR face-off: ouster. *Presented at the Autonomous Vehicle Sensors Conference 2018*.

[29] Chen H.-T., Taylor A. J., Yu N. (2016). A review of metasurfaces: physics and applications. *Rep. Prog. Phys.*.

[30] Ding F., Pors A., Bozhevolnyi S. I. (2017). Gradient metasurfaces: a review of fundamentals and applications. *Rep. Prog. Phys.*.

[31] Yu N., Capasso F. (2014). Flat optics with designer metasurfaces. *Nat. Mater.*.

[32] Shaltout A. M., Shalaev V. M., Brongersma M. L. (2019). Spatiotemporal light control with active metasurfaces. *Science*.

[33] Xie Y.-Y., Ni P.-N., Wang Q.-H. (2020). Metasurface-integrated vertical cavity surface-emitting lasers for programmable directional lasing emissions. *Nat. Nanotechnol.*.

[34] Feigenbaum E., Diest K., Atwater H. A. (2010). Unity-order index change in transparent conducting oxides at visible frequencies. *Nano Lett.*.

[35] Huang Y.-W., Lee Ho. W. H., Sokhoyan R. (2016). Gate-tunable conducting oxide metasurfaces. *Nano Lett.*.

[36] Yi F., Shim E., Zhu A. Y., Zhu H., Reed J. C., Cubukcu E. (2013). Voltage tuning of plasmonic absorbers by indium tin oxide. *Appl. Phys. Lett.*.

[37] Park J., Kang J.-H., Liu X., Brongersma M. L. (2015). Electrically tunable epsilon-near-zero (ENZ) metafilm absorbers. *Sci. Rep.*.

[38] Kim S. J., Brongersma M. L. (2016). Active flat optics using a guided mode resonance. *Opt. Lett.*.

[39] Forouzmand A., Salary M. M., Shirmanesh G. K., Sokhoyan R., Atwater H. A., Mosallaei H. (2018). Tunable all-dielectric metasurface for phase modulation of the reflected and transmitted light via permittivity tuning of indium tin oxide. *Nanophotonics*.

[40] Shirmanesh G. K., Sokhoyan R., Wu P. C., Atwater H. A. (2020). Electro-optically tunable multifunctional metasurfaces. *ACS Nano*.

[41] Park J., Jeong B. G., Lee D. (2021). All-solid-state spatial light modulator with independent phase and amplitude control for three-dimensional LiDAR applications. *Nat. Nanotechnol.*.

[42] Sabri R., Salary M. M., Mosallaei H. (2021). Quasi-static and time-modulated optical phased arrays: beamforming analysis and comparative study. *Adv. Photonics Res.*.

[43] Park J., Kang J.-H., Kim S. J., Liu X., Brongersma M. L. (2016). Dynamic reflection phase and polarization control in metasurfaces. *Nano Lett.*.

[44] Forouzmand A., Mosallaei H. (2016). Tunable two dimensional optical beam steering with reconfigurable indium tin oxide plasmonic reflectarray metasurface. *J. Opt.*.

[45] Forouzmand A., Mosallaei H. (2017). Real-time controllable and multifunctional metasurfaces utilizing indium tin oxide materials: a phased array perspective. *IEEE Trans. Nanotechnol.*.

[46] Forouzmand A., Salary M. M., Inampudi S., Mosallaei H. (2018). A tunable multigate indium-tin-oxide-assisted all-dielectric metasurface. *Adv. Opt. Mater.*.

[47] Kafaie Shirmanesh G., Sokhoyan R., Pala R. A., Atwater H. A. (2018). Dual-gated active metasurface at 1550 nm with wide (>300°) phase tunability. *Nano Lett.*.

[48] Wuttig M., Bhaskaran H., Taubner T. (2017). Phase-change materials for non-volatile photonic applications. *Nat. Photonics*.

[49] Raoux S., Xiong F., Wuttig M., Pop E. (2014). Phase change materials and phase change memory. *MRS Bull.*.

[50] Fang Z., Chen R., Zheng J., Majumdar A. (2022). Non-volatile reconfigurable silicon photonics based on phase-change materials. *IEEE J. Sel. Top. Quant. Electron.*.

[51] Zhang Q., Zhang Y., Li J., Soref R., Gu T., Hu J. (2018). Broadband nonvolatile photonic switching based on optical phase change materials: beyond the classical figure-of-merit. *Opt. Lett.*.

[52] Yin X., Steinle T., Huang L. (2017). Beam switching and bifocal zoom lensing using active plasmonic metasurfaces. Light Sci. *Appl.*.

[53] Wang Q., Maddock J., Rogers E. (2014). 1.7 Gbit/in.2 gray-scale continuous-phase-change femtosecond image storage. *Appl. Phys. Lett.*.

[54] Wang Q., Rogers E. T. F., Gholipour B. (2016). Optically reconfigurable metasurfaces and photonic devices based on phase change materials. *Nat. Photonics*.

[55] Cao T., Zheng G., Wang S., Wei C. (2015). Ultrafast beam steering using gradient Au- Ge_2_Sb_2_Te_5_ -Au plasmonic resonators. *Opt. Express*.

[56] Kats M. A., Blanchard R., Genevet P. (2013). Thermal tuning of mid-infrared plasmonic antenna arrays using a phase change material. *Opt. Lett.*.

[57] Kim Y., Wu P. C., Sokhoyan R. (2019). Phase modulation with electrically tunable vanadium dioxide phase-change metasurfaces. *Nano Lett.*.

[58] Zhu Z., Evans P. G., Haglund R. F., Valentine J. G. (2017). Dynamically reconfigurable metadevice employing nanostructured phase-change materials. *Nano Lett.*.

[59] Wu P. C., Pala R. A., Shirmanesh G. K. (2019). Dynamic beam steering with all-dielectric electro-optic III–V multiple-quantum-well metasurfaces. *Nat. Commun.*.

[60] Heni W., Kutuvantavida Y., Haffner C. (2017). Silicon–Organic and plasmonic–organic hybrid photonics. *ACS Photonics*.

[61] Burla M., Hoessbacher C., Heni W. (2019). 500 GHz plasmonic Mach–Zehnder modulator enabling sub-THz microwave photonics. *APL Photonics*.

[62] Sun X., Yu H., Deng N. (2021). Electro-optic polymer and silicon nitride hybrid spatial light modulators based on a metasurface. *Opt. Express*.

[63] Benea-Chelmus I.-C., Meretska M. L., Elder D. L., Tamagnone M., Dalton L. R., Capasso F. (2021). Electro-optic spatial light modulator from an engineered organic layer. *Nat. Commun.*.

[64] Zhang J., Kosugi Y., Otomo A. (2018). Electrical tuning of metal-insulator-metal metasurface with electro-optic polymer. *Appl. Phys. Lett.*.

[65] Thureja P., Shirmanesh G. K., Fountaine K. T., Sokhoyan R., Grajower M., Atwater H. A. (2020). Array-level inverse design of beam steering active metasurfaces. *ACS Nano*.

[66] Salary M. M., Jafar-Zanjani S., Mosallaei H. (2018). Electrically tunable harmonics in time-modulated metasurfaces for wavefront engineering. *New J. Phys.*.

[67] Sedeh H. B., Salary M. M., Mosallaei H. (2020). Time-varying optical vortices enabled by time-modulated metasurfaces. *Nanophotonics*.

[68] Lai W.-C., Chakravarty S., Zou Y., Guo Y., Chen R. T. (2013). Slow light enhanced sensitivity of resonance modes in photonic crystal biosensors. *Appl. Phys. Lett.*.

[69] Wang B., Dündar M. A., Nötzel R., Karouta F., He S., van der Heijden R. W. (2010). Photonic crystal slot nanobeam slow light waveguides for refractive index sensing. *Appl. Phys. Lett.*.

[70] Arora A., Esmaeelpour M., Bernier M., Digonnet M. J. F. (2018). High-resolution slow-light fiber Bragg grating temperature sensor with phase-sensitive detection. *Opt. Lett.*.

[71] Wülbern J. H., Petrov A., Eich M. (2009). Electro-optical modulator in a polymer-infiltrated silicon slotted photonic crystal waveguide heterostructure resonator. *Opt. Express*.

[72] Zhang X., Chung C.-J., Hosseini A. (2016). High performance optical modulator based on electro-optic polymer filled silicon slot photonic crystal waveguide. *J. Lightwave Technol.*.

[73] Gu X., Shimada T., Fuchida A., Imamura A., Matsutani A., Koyama F. (2011). Experimental demonstration of beam-steering based on slow-light waveguide amplifier. *17th Microopics Conference (MOC)*.

[74] Gu X., Shimada T., Matsutani A., Koyama F. (2012). Miniature nonmechanical beam deflector based on Bragg reflector waveguide with a number of resolution points larger than 1000. *IEEE Photonics J.*.

[75] Koyama F., Gu X. (2013). Beam steering, beam shaping, and intensity modulation based on VCSEL photonics. *IEEE J. Sel. Top. Quant. Electron.*.

[76] Kondo K., Tatebe T., Hachuda S., Abe H., Koyama F., Baba T. (2017). Fan-beam steering device using a photonic crystal slow-light waveguide with surface diffraction grating. *Opt. Lett.*.

[77] Tamanuki T., Ito H., Baba T. (2021). Thermo-optic beam scanner employing silicon photonic crystal slow-light waveguides. *J. Lightwave Technol.*.

[78] Vercruysse D., Sapra N. V., Yang K. Y., Vučković J. (2021). Inverse-designed photonic crystal circuits for optical beam steering. *ACS Photonics*.

[79] Ito H., Kusunoki Y., Maeda J. (2020). Wide beam steering by slow-light waveguide gratings and a prism lens. *Optica*.

[80] Fuchida A., Matsutani A., Ahmed M., Bakry A., Koyama F. (2014). Low-polarization dependent thermo-optic phase-shift in slow light Bragg reflector waveguide for beam steering and optical switching. *Jpn. J. Appl. Phys.*.

[81] Joannopoulos J. D., Johnson S. G., Winn J. N., Meade R. D. (2011). *Photonic Crystals: Molding the Flow of Light –*.

[82] Sakoda K. (2013). *Optical Properties of Photonic Crystals*.

[83] Abe H., Takeuchi M., Kondo K. (2018). Two-dimensional beam-steering device using a doubly periodic Si photonic-crystal waveguide. *Opt. Express*.

[84] Takeuchi G., Terada Y., Takeuchi M., Abe H., Ito H., Baba T. (2018). Thermally controlled Si photonic crystal slow light waveguide beam steering device. *Opt. Express*.

[85] Maeda J., Akiyama D., Ito H., Abe H., Baba T. (2019). Prism lens for beam collimation in a silicon photonic crystal beam-steering device. *Opt. Lett.*.

[86] Ito H., Tatebe T., Abe H., Baba T. (2018). Wavelength-division multiplexing Si photonic crystal beam steering device for high-throughput parallel sensing. *Opt. Express*.

[87] Tetsuya R., Abe H., Ito H., Baba T. (2019). Efficient light transmission, reception and beam forming in photonic crystal beam steering device in a phased array configuration. *Jpn. J. Appl. Phys.*.

[88] Acoleyen K. V., Bogaerts W., Jágerská J., Thomas N. L., Houdré R., Baets R. (2009). Off-chip beam steering with a one-dimensional optical phased array on silicon-on-insulator. *Opt. Lett.*.

[89] Sun J., Timurdogan E., Yaacobi A., Hosseini E. S., Watts M. R. (2013). Large-scale nanophotonic phased array. *Nature*.

[90] Hutchison D. N., Sun J., Doylend J. K. (2016). High-resolution aliasing-free optical beam steering. *Optica*.

[91] Hulme J. C., Doylend J., Heck M. (2015). Fully integrated hybrid silicon two dimensional beam scanner. *Opt. Express*.

[92] Chung S., Abediasl H., Hashemi H. (2018). A monolithically integrated large-scale optical phased array in silicon-on-insulator CMOS. *IEEE J. Solid State Circ.*.

[93] Phare C. T., Shin M. C., Sharma J. (2018). Silicon optical phased array with grating lobe-free beam formation over 180 degree field of view. *Conference on Lasers and Electro-Optics (2018), paper SM3I.2*.

[94] Komma J., Schwarz C., Hofmann G., Heinert D., Nawrodt R. (2012). Thermo-optic coefficient of silicon at 1550 nm and cryogenic temperatures. *Appl. Phys. Lett.*.

[95] Xiao F., Hu W., Xu A. (2005). Optical phased-array beam steering controlled by wavelength. *Appl. Opt.*.

[96] Hosseini A., Kwong D., Zhao Y. (2009). Unequally spaced waveguide arrays for silicon nanomembrane-based efficient large angle optical beam steering. *IEEE J. Sel. Top. Quant. Electron.*.

[97] Kwong D., Hosseini A., Zhang Y., Chen R. T. (2011). 1 × 12 Unequally spaced waveguide array for actively tuned optical phased array on a silicon nanomembrane. *Appl. Phys. Lett.*.

[98] Doylend J. K., Heck M. J. R., Bovington J. T., Peters J. D., Coldren L. A., Bowers J. E. (2011). Two-dimensional free-space beam steering with an optical phased array on silicon-on-insulator. *Opt. Express*.

[99] Kwong D., Hosseini A., Covey J. (2014). On-chip silicon optical phased array for two-dimensional beam steering. *Opt. Lett.*.

[100] Yaacobi A., Sun J., Moresco M., Leake G., Coolbaugh D., Watts M. R. (2014). Integrated phased array for wide-angle beam steering. *Opt. Lett.*.

[101] Aflatouni F., Abiri B., Rekhi A., Hajimiri A. (2015). Nanophotonic projection system. *Opt. Express*.

[102] Abediasl H., Hashemi H. (2015). Monolithic optical phased-array transceiver in a standard SOI CMOS process. *Opt. Express*.

[103] Poulton C. V., Byrd M. J., Raval M. (2017). Large-scale silicon nitride nanophotonic phased arrays at infrared and visible wavelengths. *Opt. Lett.*.

[104] Komljenovic T., Helkey R., Coldren L., Bowers J. E. (2017). Sparse aperiodic arrays for optical beam forming and LIDAR. *Opt. Express*.

[105] Song W., Gatdula R., Abbaslou S. (2015). High-density waveguide superlattices with low crosstalk. *Nat. Commun.*.

[106] Ashtiani F., Aflatouni F. (2019). *N* × *N* optical phased array with 2*N* phase shifters. *Opt. Express*.

[107] Fatemi R., Khachaturian A., Hajimiri A. (2019). A nonuniform sparse 2-D large-FOV optical phased array with a low-power PWM drive. *IEEE J. Solid State Circ.*.

[108] Shin M. C., Mohanty A., Watson K. (2020). Chip-scale blue light phased array. *Opt. Lett.*.

[109] Dostart N., Zhang B., Khilo A. (2020). Serpentine optical phased arrays for scalable integrated photonic lidar beam steering. *Optica*.

[110] Poulton C. V., Byrd M. J., Russo P. (2019). Long-range LiDAR and free-space data communication with high-performance optical phased arrays. *IEEE J. Sel. Top. Quant. Electron.*.

[111] Kim S.-H., You J.-B., Ha Y.-Gi. (2019). Thermo-optic control of the longitudinal radiation angle in a silicon-based optical phased array. *Opt. Lett.*.

[112] Tyler N. A., Fowler D., Malhouitre S. (2019). SiN integrated optical phased arrays for two-dimensional beam steering at a single near-infrared wavelength. *Opt. Express*.

[113] Zhang Y., Ling Yi-C., Zhang K. (2019). Sub-wavelength-pitch silicon-photonic optical phased array for large field-of-regard coherent optical beam steering. *Opt. Express*.

[114] Miller S. A., Chang Y.-C., Phare C. T. (2020). Large-scale optical phased array using a low-power multi-pass silicon photonic platform. *Optica*.

[115] Moffet A. (1968). Minimum-redundancy linear arrays. *IEEE Trans. Antenn. Propag.*.

[116] Poulton C. V., Yaacobi A., Cole D. B. (2017). Coherent solid-state LIDAR with silicon photonic optical phased arrays. *Opt. Lett.*.

[117] Kim T., Bhargava P., Poulton C. (2019). A single-chip optical phased array in a wafer-scale silicon photonics/CMOS 3D-integration platform. *IEEE J. Solid State Circ.*.

[118] Pan G., Xu C., Xie Y. (2019). Ultra-compact electrically controlled beam steering chip based on coherently coupled VCSEL array directly integrated with optical phased array. *Opt. Express*.

[119] Sayyah K., Efimov O. M., Patterson P. (2015). Two-dimensional pseudo-random optical phased array based on tandem optical injection locking of vertical cavity surface emitting lasers. *Opt. Express*.

[120] Yoo B.-W., Megens M., Chan T. (2013). Optical phased array using high contrast gratings for two dimensional beamforming and beamsteering. *Opt. Express*.

[121] Chan T. K., Megens M., Yoo B.-W. (2013). Optical beamsteering using an 8 × 8 MEMS phased array with closed-loop interferometric phase control. *Opt. Express*.

[122] Yang W., Tianbo S., Rao Y. (2014). High speed optical phased array using high contrast grating all-pass filters. *Opt. Express*.

[123] Molesky S., Lin Z., Piggott A. Y., Jin W., Vucković J., Rodriguez A. W. (2018). Inverse design in nanophotonics. *Nat. Photonics*.

[124] Haffner C., Heni W., Elder D. L. (2017). Harnessing nonlinearities near material absorption resonances for reducing losses in plasmonic modulators. *Opt. Mater. Express*.

[125] Haffner C., Chelladurai D., Fedoryshyn Y. (2018). Low-loss plasmon-assisted electro-optic modulator. *Nature*.

[126] Balli F., Sultan M. A., Ozdemir A., Hastings J. T. (2021). An ultrabroadband 3D achromatic metalens. *Nanophotonics*.

[127] Sultan M. A., Balli F., Lau D. L., Hastings J. T. (2021). Hybrid metasurfaces for simultaneous focusing and filtering. *Opt. Lett.*.

